# Supplementary material for: Recombination shapes the dynamics of selective constraint in global populations of human gut bacteria
Source: PLoS Biol. 2026 Oct 12;24(10):e3004052. doi: 10.1371/journal.pbio.3004052 (PMC13690274; doi:10.1371/journal.pbio.3004052)
Supplement: Supplementary file 1 [file pbio.3004052.s001.pdf]

S1 Text: Supplemental Figures

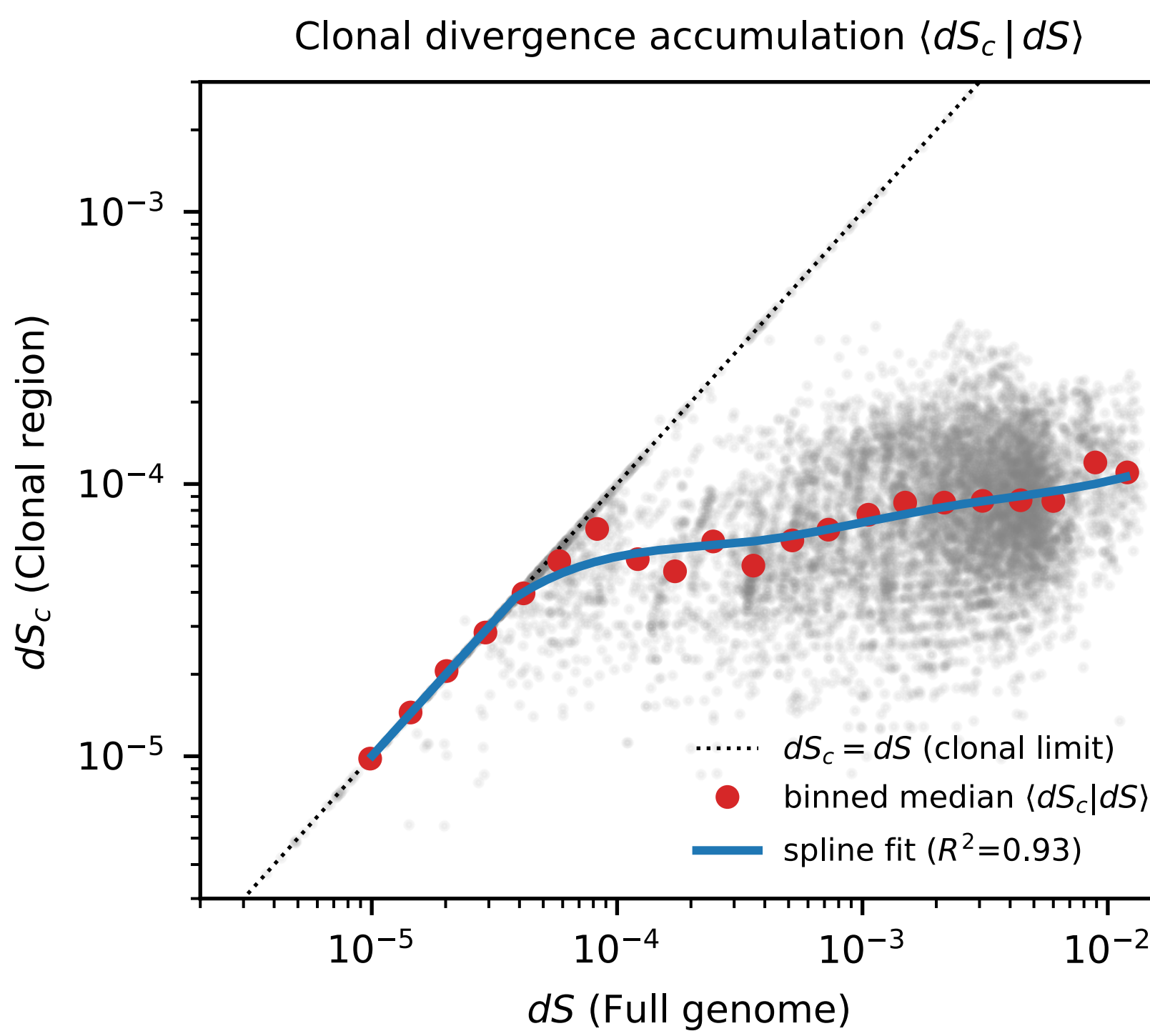

Fig A. Empirical fit of clonal versus total synonymous divergence. Grey points show the clonal synonymous divergence ($d_S^{(c)}$) for each of the strain pairs in Fig 2B. The dashed line denotes the clonal limit ($d_S^{(c)} = d_S$) expected in the absence of recombination, while red points denote the median values of $d_S^{(c)}$ in sliding windows of d_S. These data show that the total d_S between many strain pairs is dominated by recombined segments (34). The blue solid line shows a spline fit to the red points, which was used to parameterize the phenomenological model in Fig 2B (Methods Section 3). The data and code needed to generate this figure can be found in `https://doi.org/10.5281/zenodo.22837797`.

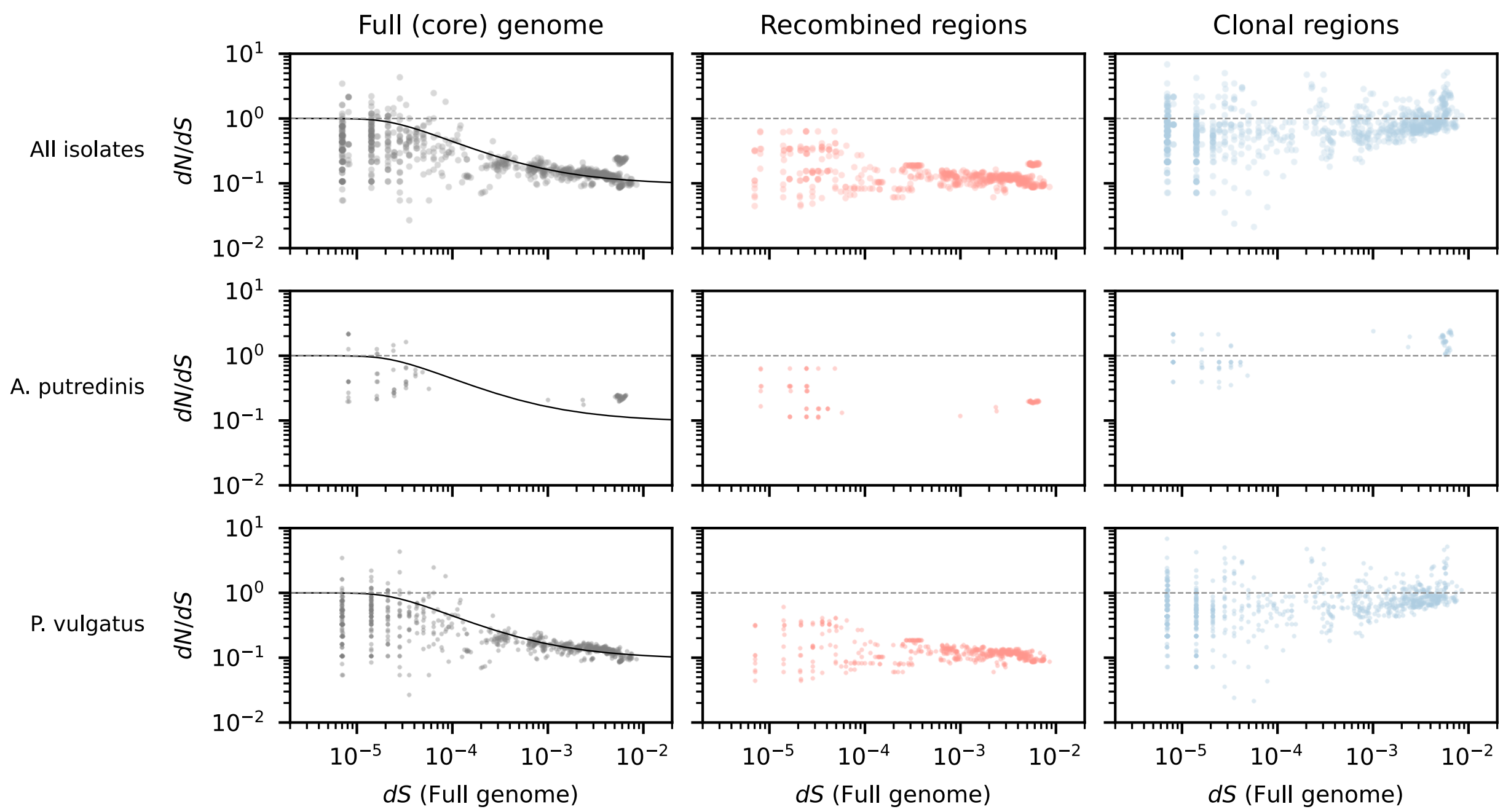

Fig B. Analogous versions of Fig 2 for a collection of *Alistipes putredinis* and *Phocaeicola vulgatus* isolates. dN/dS dynamics estimated from pairs of cultured isolate genomes (41 *A. putredinis* and 369 *P. vulgatus* assemblies, each mapped to the corresponding MIDAS reference genome), with recombined regions detected per pair by the close-pair HMM (34). The top row pools all isolate pairs across both species; the lower rows show *A. putredinis* and *P. vulgatus* separately. Columns follow Fig 2: (left) genome-wide dN/dS across strain pairs as a function of synonymous divergence dS, estimated from 1D and 4D sites, with the neutral recombination expectation (black curve); (middle) dN/dS computed only within CP-HMM–detected recombined regions; (right) dN/dS within the remaining clonal regions. The dashed line marks $dN/dS = 1$. The data and code needed to generate this figure can be found in `https://doi.org/10.5281/zenodo.22837797`.

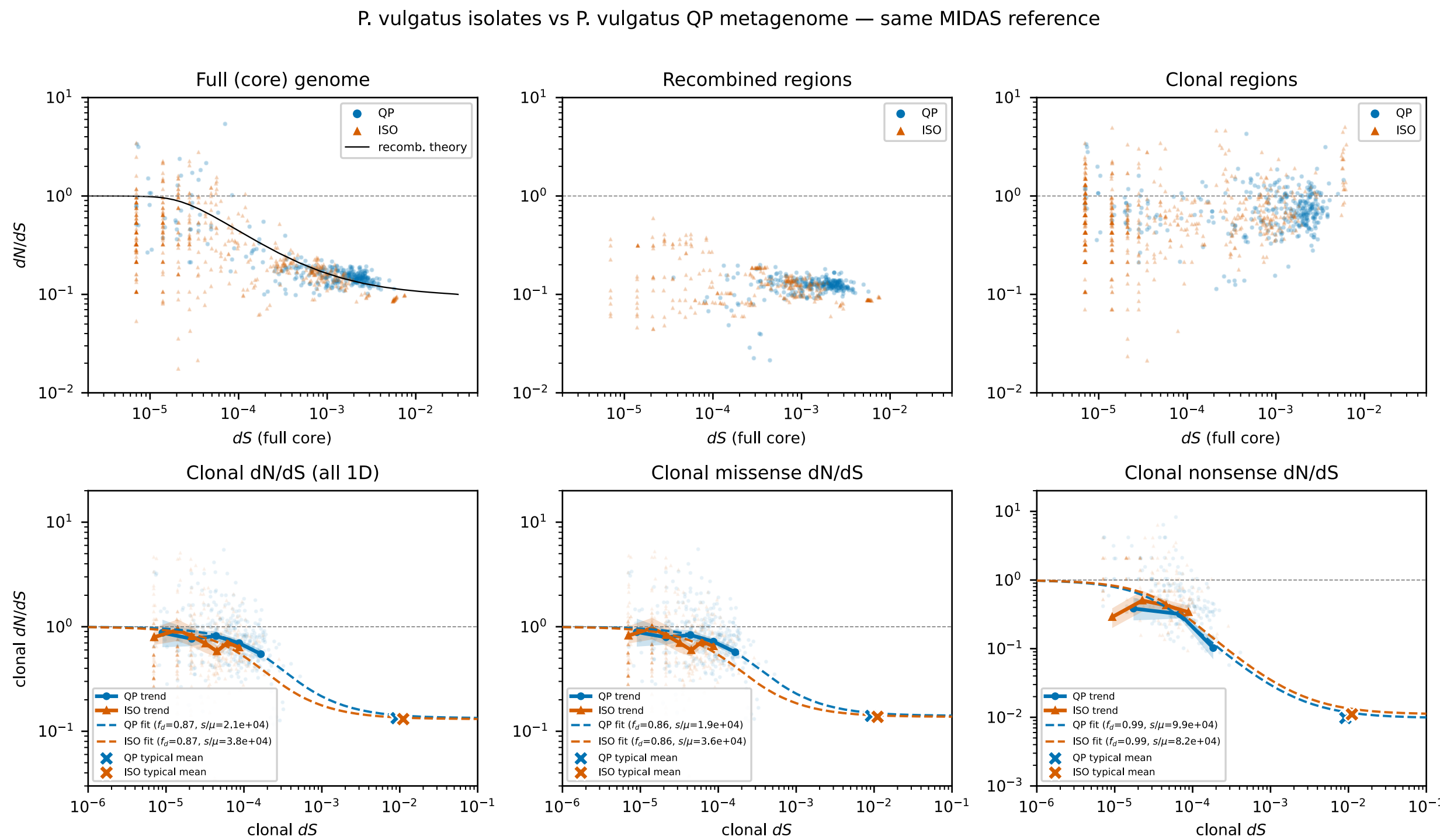

Fig C. Estimated dN/dS dynamics in *P. vulgatus* are consistent across metagenomic and isolate datasets. Points show a direct comparison between the estimated dN/dS levels in *P. vulgatus* (formerly *Bacteroides vulgatus*) for the quasi-phased metagenomic cohort in Fig 2B (QP) and the isolate collection in Fig B in S1 Text (ISO), aligned to the same MIDAS reference genome (Methods Section 9). Top row: dN/dS versus dS for the full (core) genome, the recombined regions, and the clonal regions of each strain pair, overlaying ISO (triangles) and QP cohorts (circles); the black curve in the left panel shows the same phenomenological recombination model as Fig 2B. Bottom row: decay of clonal dN/dS versus clonal dS, separated into all nonsynonymous (1D), missense, and nonsense mutations. Shaded points denote individual pairs; thick lines and shaded bands are bootstrap medians and 95% confidence intervals over adaptive logarithmic bins (targeting a fixed number of pairs per bin); dashed lines are per-class purifying-selection fits, with the fraction of constrained sites f_d fixed from the unrelated-pair equilibrium and s/μ fit by weighted least squares. The "x" markers show the unrelated-pair average, which anchors the high-dS equilibrium. Note that the QP cohort bins two clades (*P. vulgatus* and *P. dorei*) onto the shared *P. vulgatus* reference; only within-clade pairs are retained for QP, and isolate close pairs are restricted to identical fraction > 0.75 to match the QP clonal-fraction cutoff. The dashed grey line marks $dN/dS = 1$. The data and code needed to generate this figure can be found in `https://doi.org/10.5281/zenodo.22837797`.

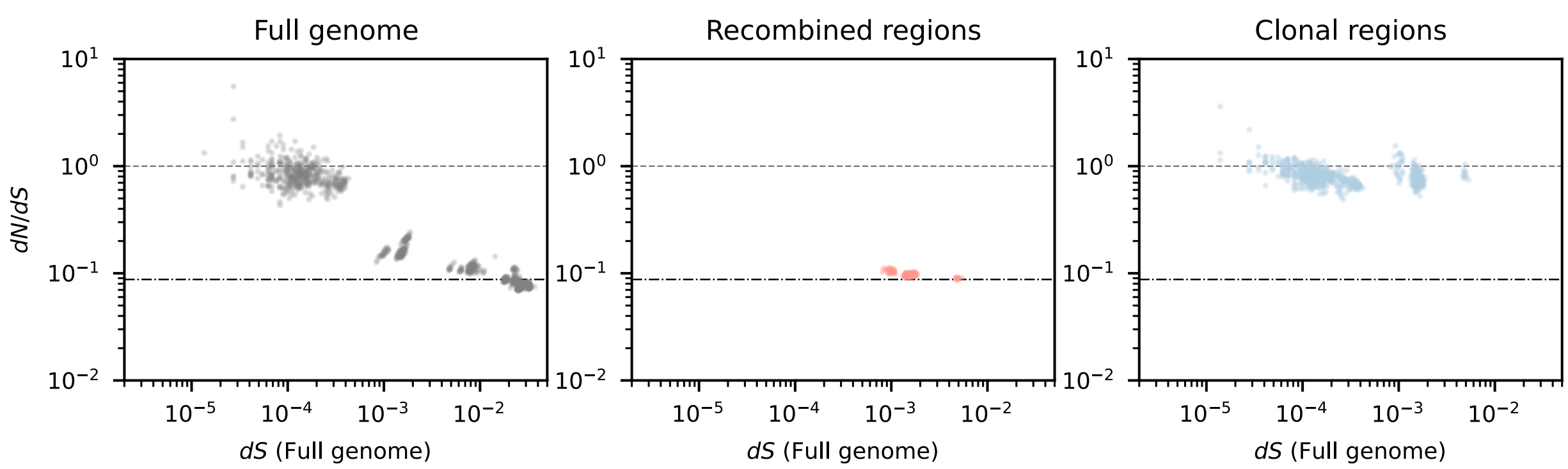

Fig D. Analogous version of Fig 2 for a collection of *Staphylococcus aureus* strains. Left: Genome-wide dN/dS dynamics across pairs of *S. aureus* strains, estimated from 1D and 4D sites. Middle: dN/dS values computed only within recombined regions corresponding to the large recombination events in ST34, ST239, and ST582 (Methods Section 8). Right: dN/dS values computed within the remaining clonal regions. The data and code needed to generate this figure can be found in `https://doi.org/10.5281/zenodo.22837797`.

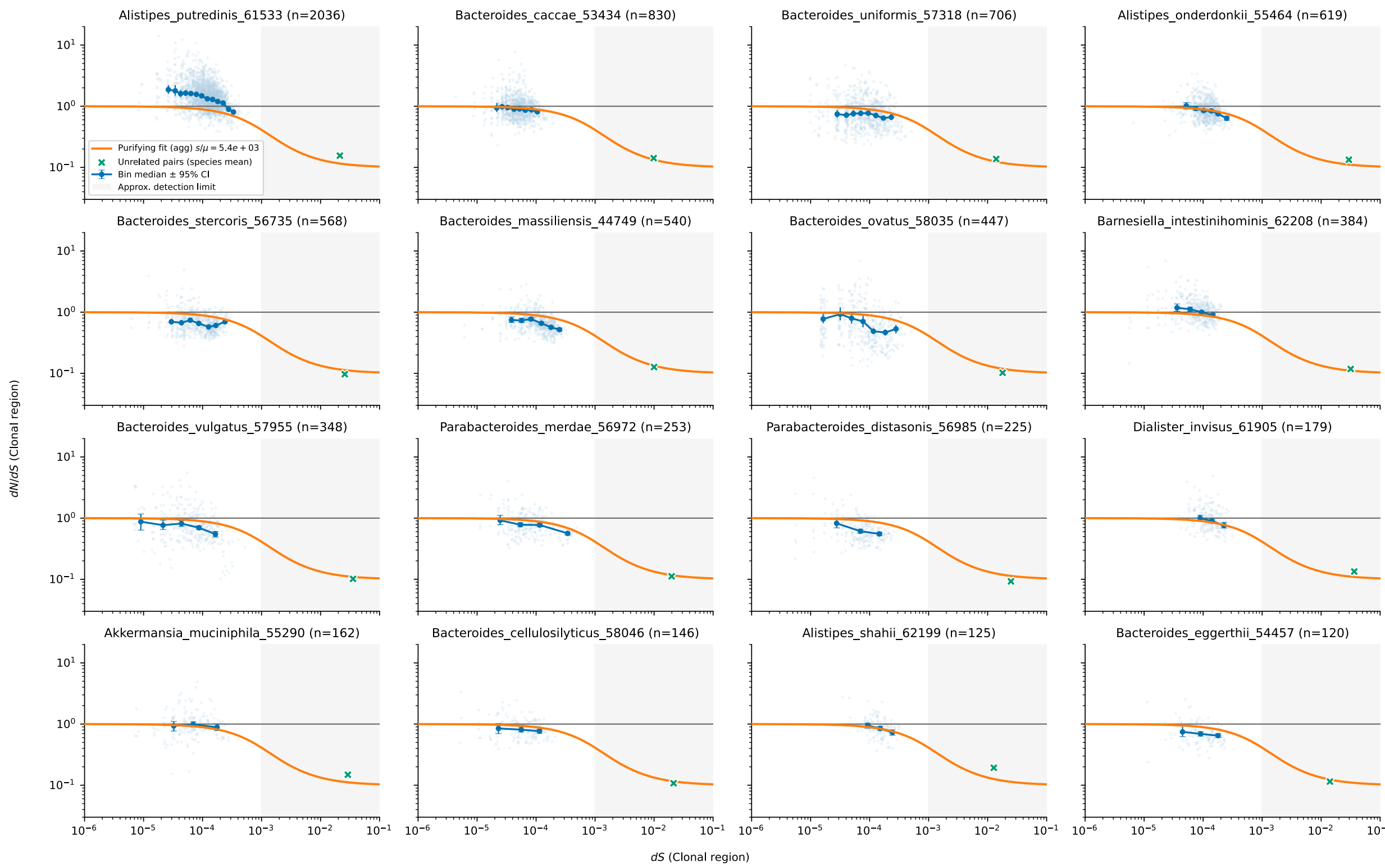

Fig E. Species-level clonal d_N/d_S dynamics (all nonsynonymous sites). Light-blue points show pairwise clonal d_N/d_S versus clonal d_S estimated by Poisson-thinning; dark-blue markers with error bars denote the median of each divergence bin with 95% CIs from pooled counts. The orange curve is the same aggregate two-class purifying-selection fit used in Fig 3, drawn identically in every panel for reference. Green "X" marks indicate the mean values of d_N/d_S and d_S between unrelated, fully recombined pairs for that species; the gray line marks $d_N/d_S = 1$; the shaded band denotes the approximate detection limit ($d_S \gtrsim 10^{-3}$). Species and bins with too few pairs are omitted; panels are ordered by the number of available pairs in each species. The data and code needed to generate this figure can be found in `https://doi.org/10.5281/zenodo.22837797`.

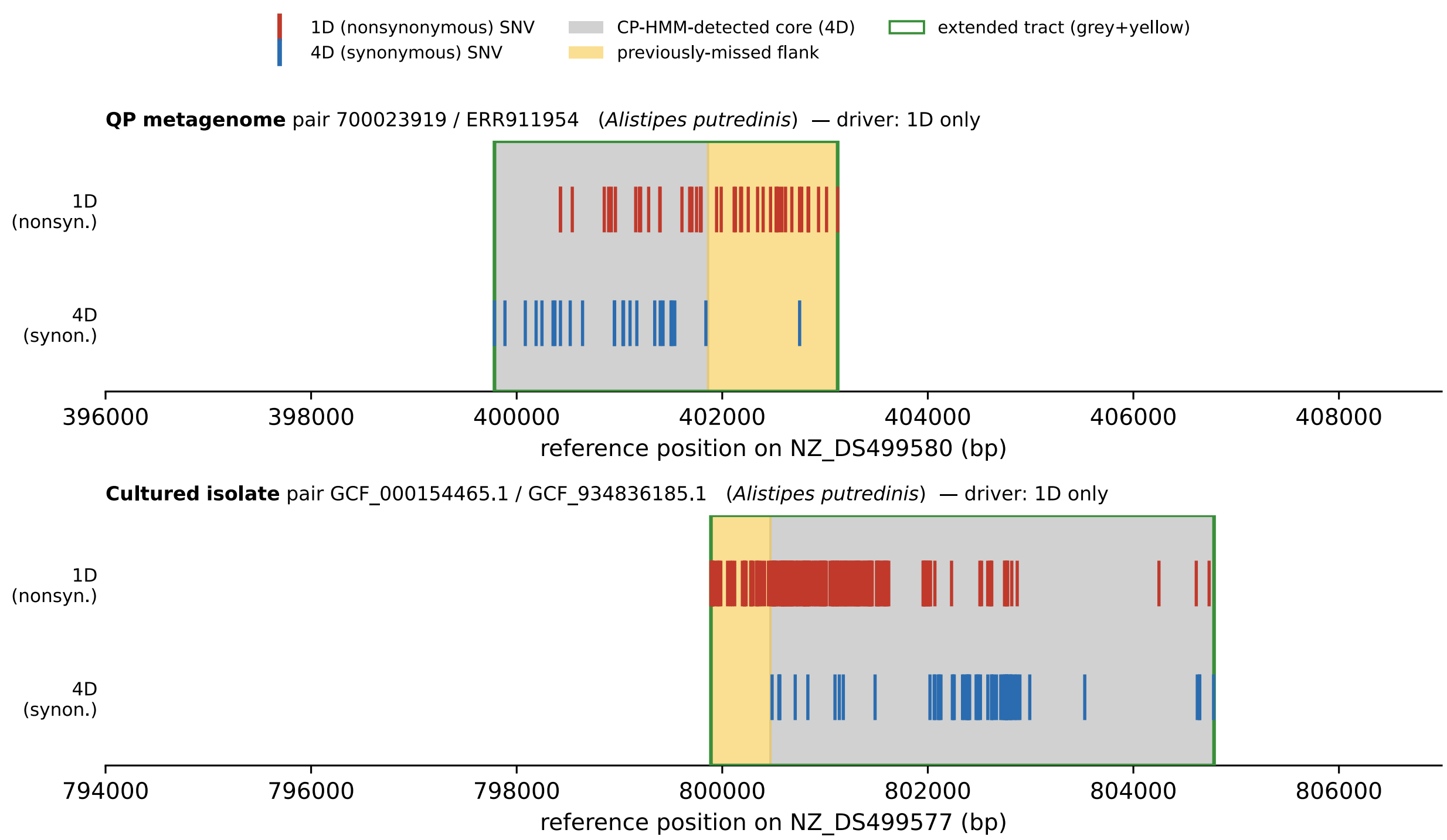

Fig F. Recombination tracts with nonsynonymous-rich boundaries can be missed by synonymous-only detection. Examples are shown from two example closely related strain pairs from *Alistipes putredinis*: one from the original metagenomic cohort in Fig 2 (top) and one from the set of cultured isolates in Fig B in S1 Text (bottom). Each tick marks a pairwise SNV at a nonsynonymous (1D, red) or synonymous (4D, blue) site. Because our CP-HMM method detects transfers from 4D SNV density alone, it captures the tract core (grey, dense in both classes) but truncates where the 4D signal fades, leaving an adjacent flank (yellow) that is dense in 1D yet nearly devoid of 4D differences, which is misclassified as part of the clonal frame. Extending the boundary over this 1D-dense flank (green outline) reassigns the stranded nonsynonymous differences to the recombined region. Such 1D-rich tract boundaries (which in this case overlap with a natural-competence gene *comEC/rec2* in the top panel and an alpha-glucan phosphorylase in the bottom panel) are invisible to any 4D-based detection method, and can spuriously inflate the clonal d_N/d_S if left uncorrected. The data and code needed to generate this figure can be found in `https://doi.org/10.5281/zenodo.22837797`.

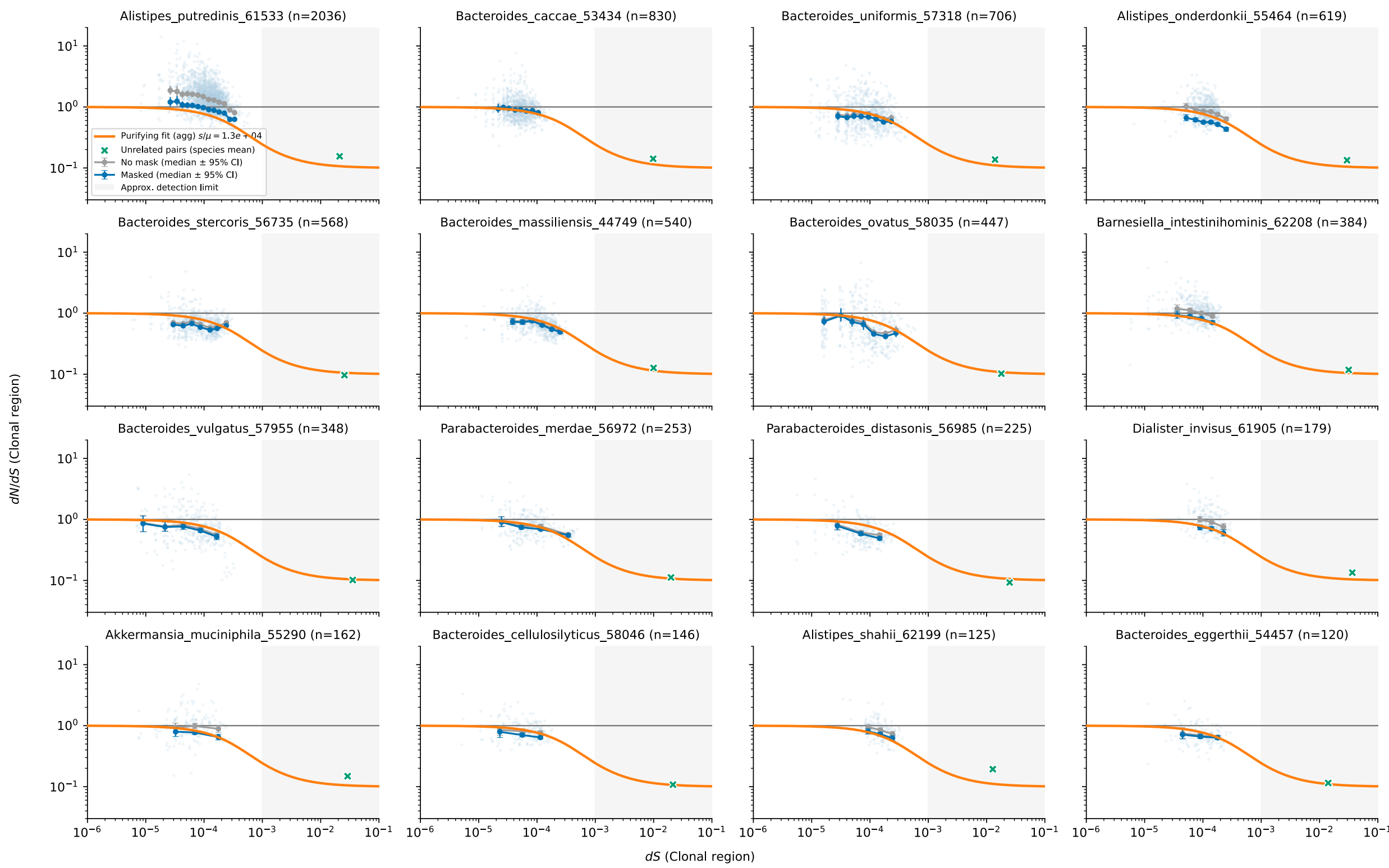

Fig G. Modified clonal d_N/d_S dynamics after extending recombination boundaries to account for nonsynonymous-rich flanks. Analogous version of Fig E in S1 Text computed for the extended recombination tract boundaries identified using the heuristic approach described in Methods Section 10. The corrections are concentrated in *Alistipes* (particularly *A. putredinis*) and are mostly negligible in *Bacteroides*. These results suggest that the elevated clonal d_N/d_S signal in a handful of species is driven by genuine recombination events at nonsynonymous-rich loci that our synonymous-based detection method misses. The data and code needed to generate this figure can be found in `https://doi.org/10.5281/zenodo.22837797`.

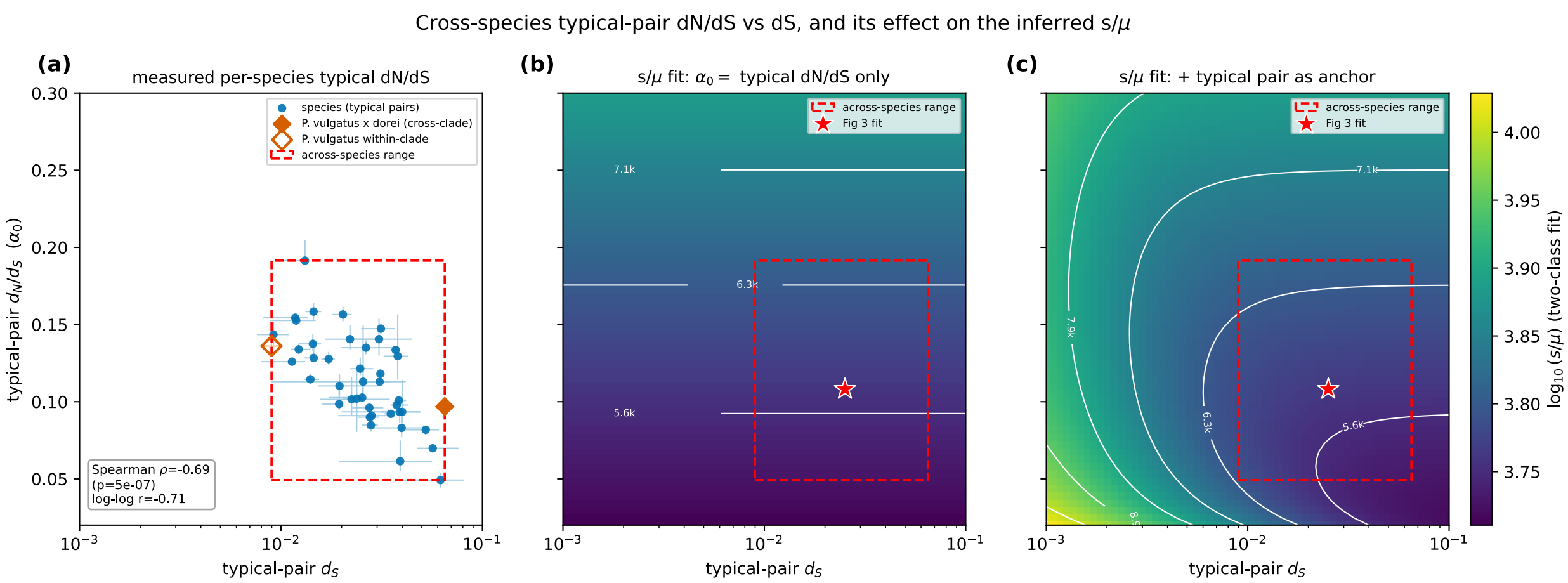

Fig H. Cross-species variation in typical-pair d_N/d_S and its effect on the inferred strength of purifying selection. (a) Points show a zoomed in version of the green crosses in Fig 3, showing the mean dN/dS values for fully recombined strain pairs for each species. Error bars depict the central 80% (16–84%) inter-pair spread for each species. Orange diamonds mark *P. vulgatus* within-clade (open) and cross-clade *vulgatus*$\times$*dorei* (filled) pairs, while the dashed red box marks the full across-species range. These data show that the average d_N/d_S value of each species exhibits a small but significant decline with $\overline{d_S}$ (Spearman $\rho = -0.69$, $p = 5 \times 10^{-7}$), consistent with a portion of extremely weakly selected mutations that continue to be purged at these long timescales. (b,c) Refitted clonal selection strength s_1/μ [Eq. 13, Methods Section 4] as the anchored value of $\overline{d_N}/\overline{d_S} \equiv \alpha_0$ and $\overline{d_S}$ are swept over the same plane. Red star denotes the average value used in Fig 3. As in the main text, our fitting procedure in panel b only uses the intercept α_0, so s_1/μ is independent of $\overline{d_S}$. In panel c, the value of $\overline{d_S}$ is included as an additional datapoint. Across the entire range of species-specific dN/dS values, the estimated value of s_1/μ varies from ~ 5–8×10^3, showing that the inferred strength of purifying selection is robust to the observed cross-species spread in $\overline{d_N}/\overline{d_S}$. The data and code needed to generate this figure can be found in `https://doi.org/10.5281/zenodo.22837797`.

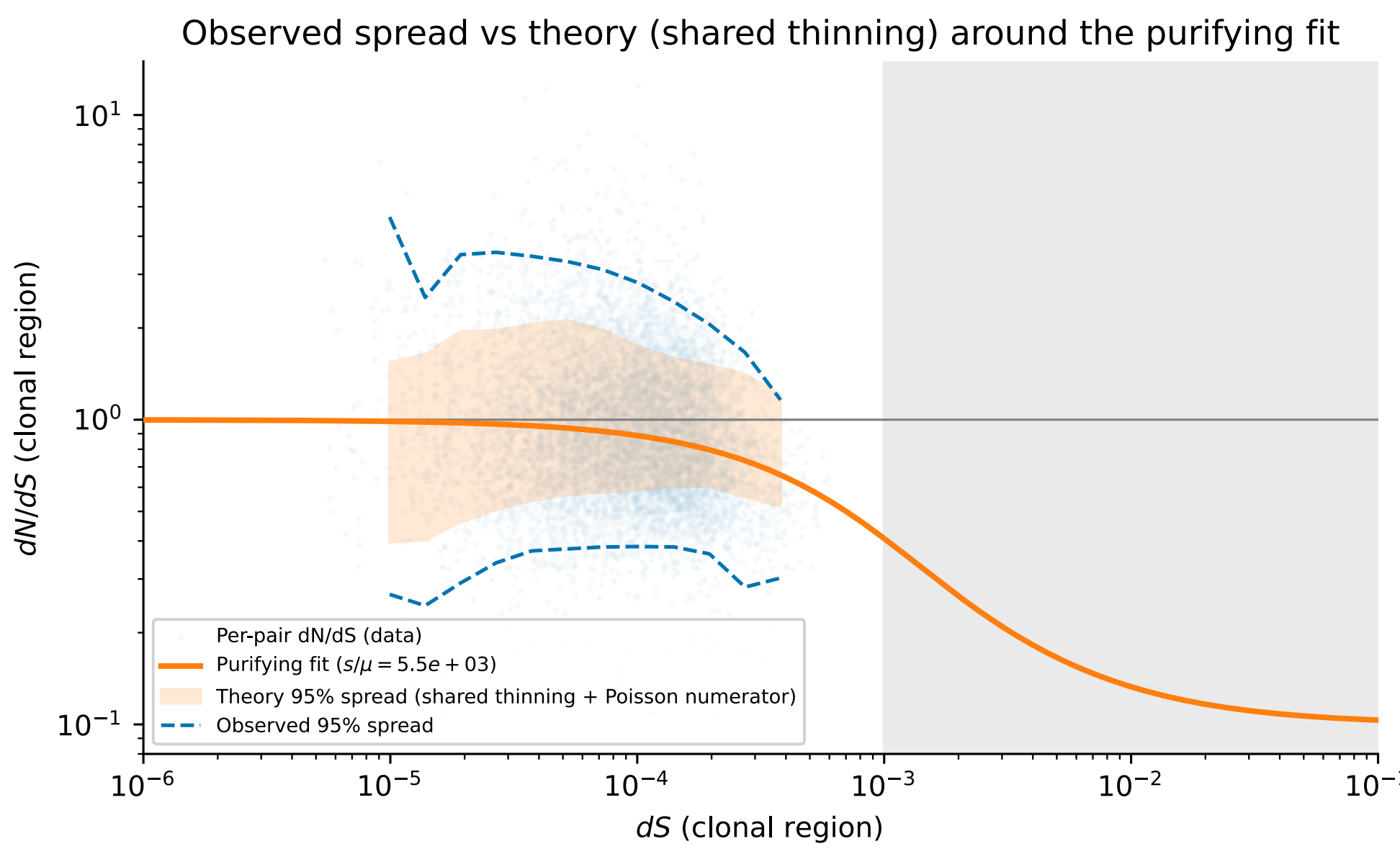

Fig I. Quantifying the spread of clonal dN/dS compared to the purifying-selection null model. Dashed line denotes the estimated 95% spread for the observed data (points), while the shaded band denotes the predicted 95% spread of the fitted purifying selection model (solid line) from finite sampling alone (Methods Section 2). The observed scatter is only ~ 1.5–$1.8\times$ wider than this sampling-only model predicts. The data and code needed to generate this figure can be found in `https://doi.org/10.5281/zenodo.22837797`.

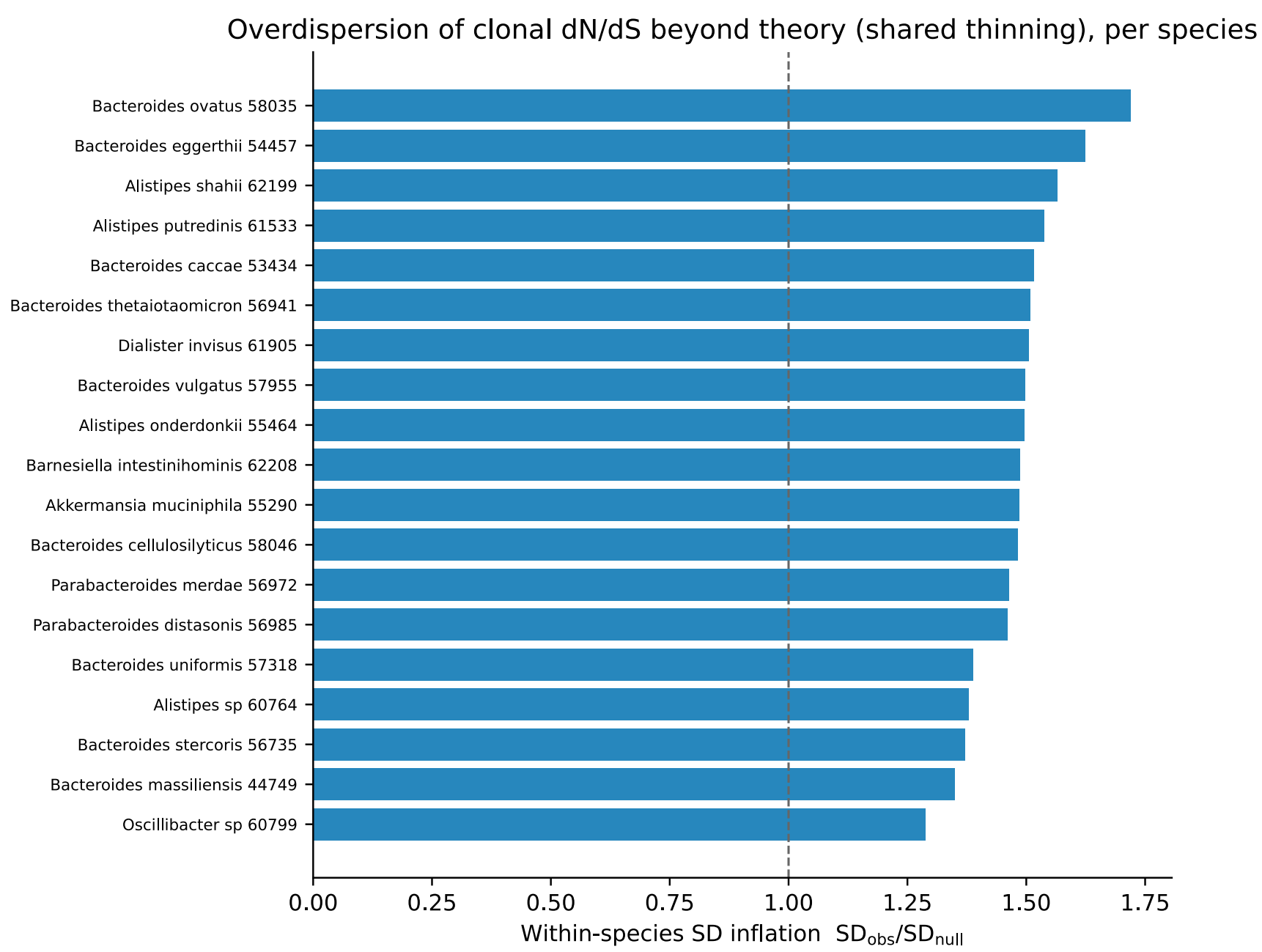

Fig J. Within-species overdispersion of clonal dN/dS. An analogous version of Fig I, computed for individual species. For each species, bars show the ratio of the observed to the model-predicted spread of d_N/d_S ($\mathrm{SD}_{\mathrm{obs}}/\mathrm{SD}_{\mathrm{null}}$), log scale; dashed line at 1 marks pure sampling). The excess is modest (~ 1.3–$1.7\times$) and similar across species. The data and code needed to generate this figure can be found in `https://doi.org/10.5281/zenodo.22837797`.

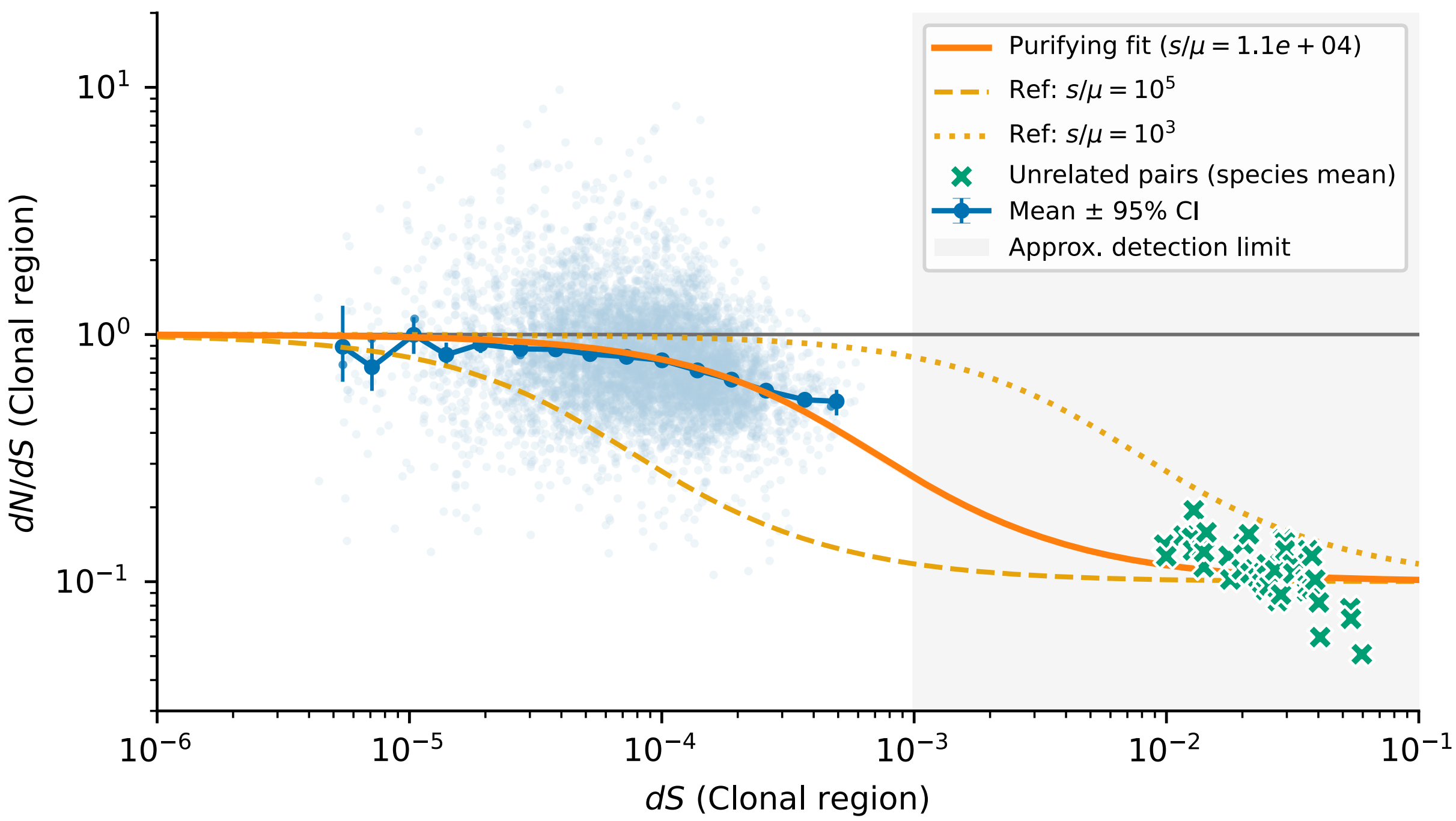

Fig K. An analogous version of Fig 3 excluding *Alistipes putredinis*. The overall results remain unchanged, with only a slightly higher estimated selection coefficient. The data and code needed to generate this figure can be found in `https://doi.org/10.5281/zenodo.22837797`.

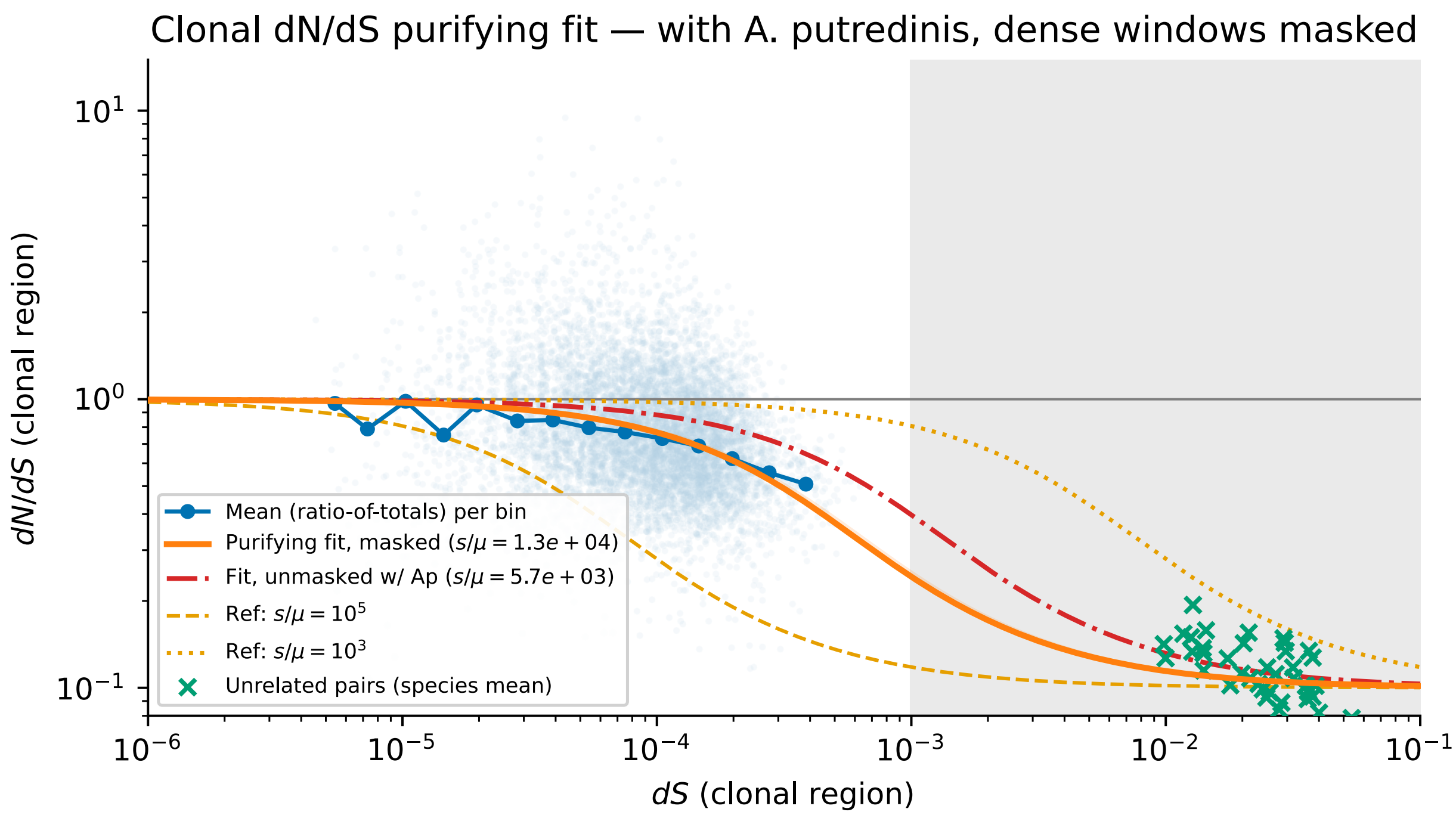

Fig L. An analogous version of Fig 3 computed from the extended recombination tract boundaries. When the nonsynonymous rich edges are reassigned to the recombined region, the pooled estimate of s/μ (including *A. putredinis*) rises to roughly twice the main-text value (red curve), placing it close to the no-*Ap* fit in Fig K in S1 Text ($s/\mu \approx 1.1 \times 10^4$). The effective fitness cost varies by about twofold under these different estimation methods, confirming that its order of magnitude is robust to the correction. The data and code needed to generate this figure can be found in `https://doi.org/10.5281/zenodo.22837797`.

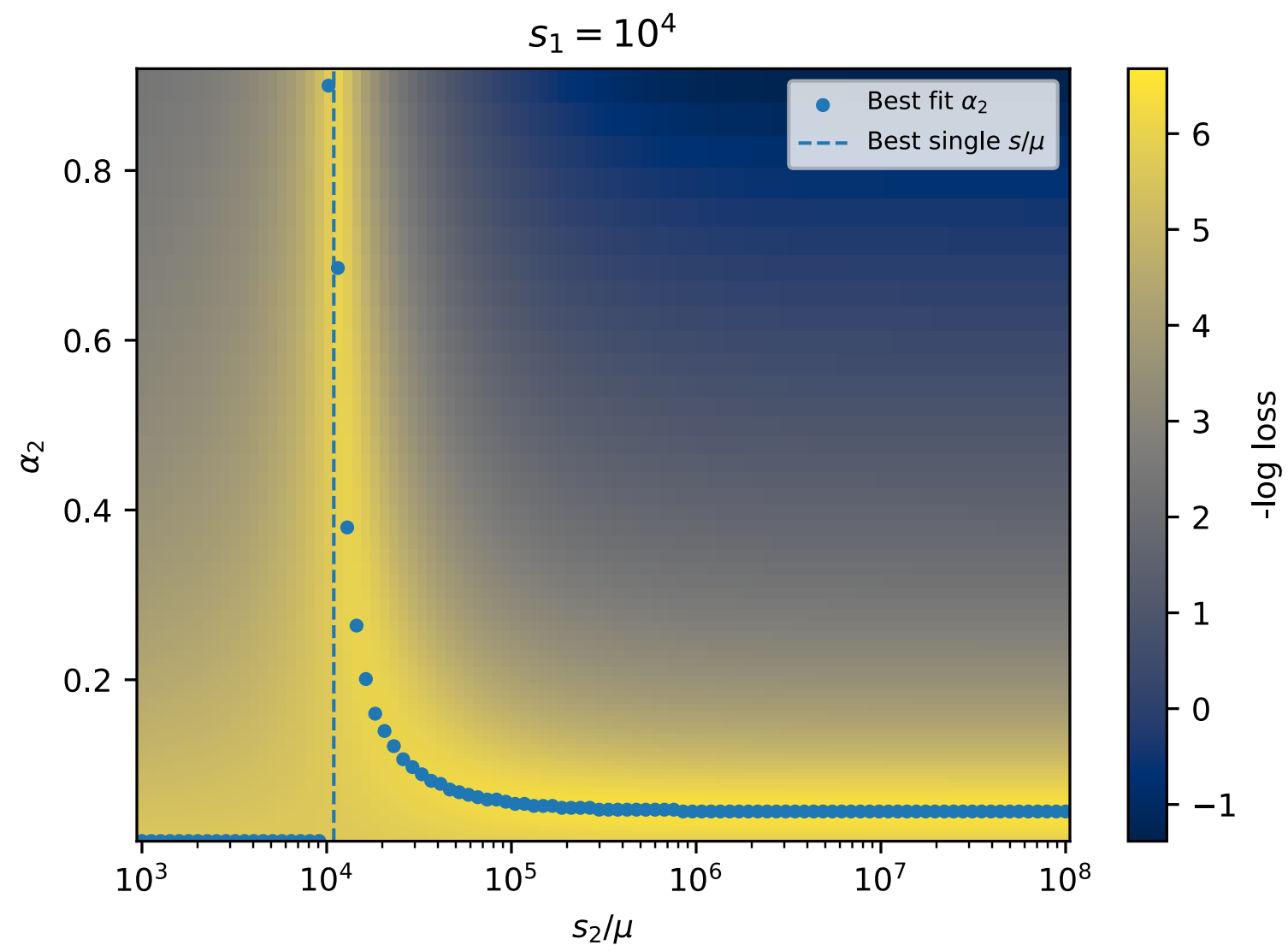

Fig M. Loss surface of the three-class purifying selection model in Methods Section 4. Each pixel shows the model fit quality ($-\log(\mathrm{wMSE})$, Eq. 17) as a function of the fraction of strongly deleterious sites (α_2) and their scaled selection coefficient (s_2/μ). Models were fit to all nonsynonymous sites, excluding *Alistipes putredinis*. The loss surface was evaluated by varying α_2 and s_2/μ, with $\alpha_0 = 0.1$ and $s_1/\mu = 10^4$ held fixed at the values obtained from the best-fit two-class model. The dashed vertical line marks the best-fit single-class value of s/μ, while dots indicate the best-fit α_2 value at each value of s_2/μ. The shape of the loss surface suggests that introducing an additional strongly deleterious mutation class ($s_2/\mu \gtrsim 10^5$) modestly improves the fit, but the inferred fraction of such sites remains very small ($\alpha_2 < 0.05$). The data and code needed to generate this figure can be found in `https://doi.org/10.5281/zenodo.22837797`.

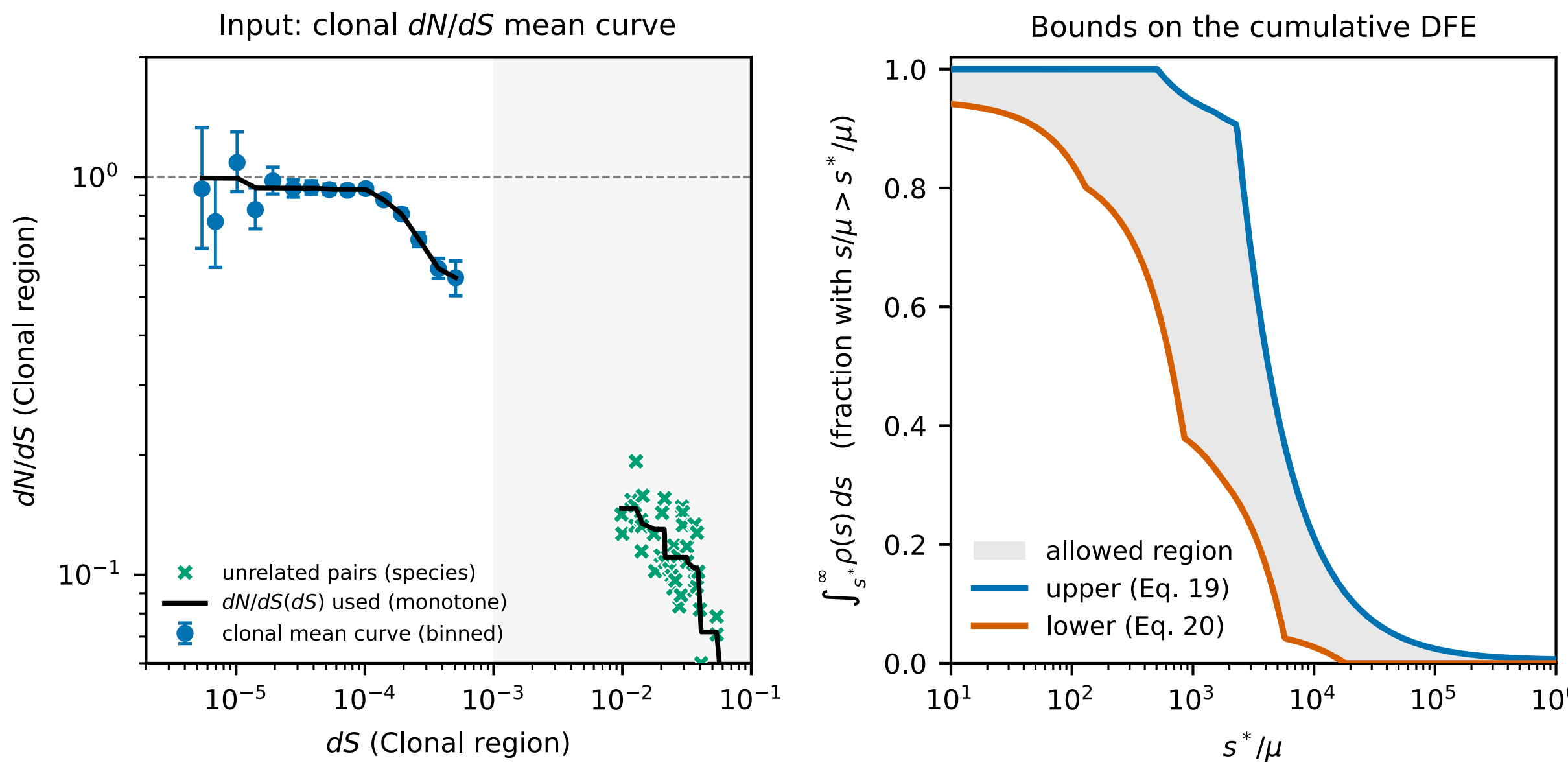

Fig N. Constraints on the distribution of fitness effects in the purifying selection model. Illustration of the bounds on the full distribution of fitness costs $\rho(s)$ described in Eqns. 19 and 20 in Methods Section 4. (a) The clonal dN/dS values in Fig 3 were first fit to a monotonically declining function (black line). (b) We then used this inferred dN/dS curve to estimate bounds on the reverse CDF of $\rho(s)$ at different values of s^*. Top line shows an upper bound obtained by taking the minimum value of Eq. 19 over all values of d_S at a given value of s^*. Bottom line shows a corresponding lower bound by taking the maximum value of Eq. 20 over all values of d_S. The shaded region between these two bounds denotes the range of allowable values of $\int_{s^*}^{\infty} \rho(s)ds$. The data and code needed to generate this figure can be found in `https://doi.org/10.5281/zenodo.22837797`.

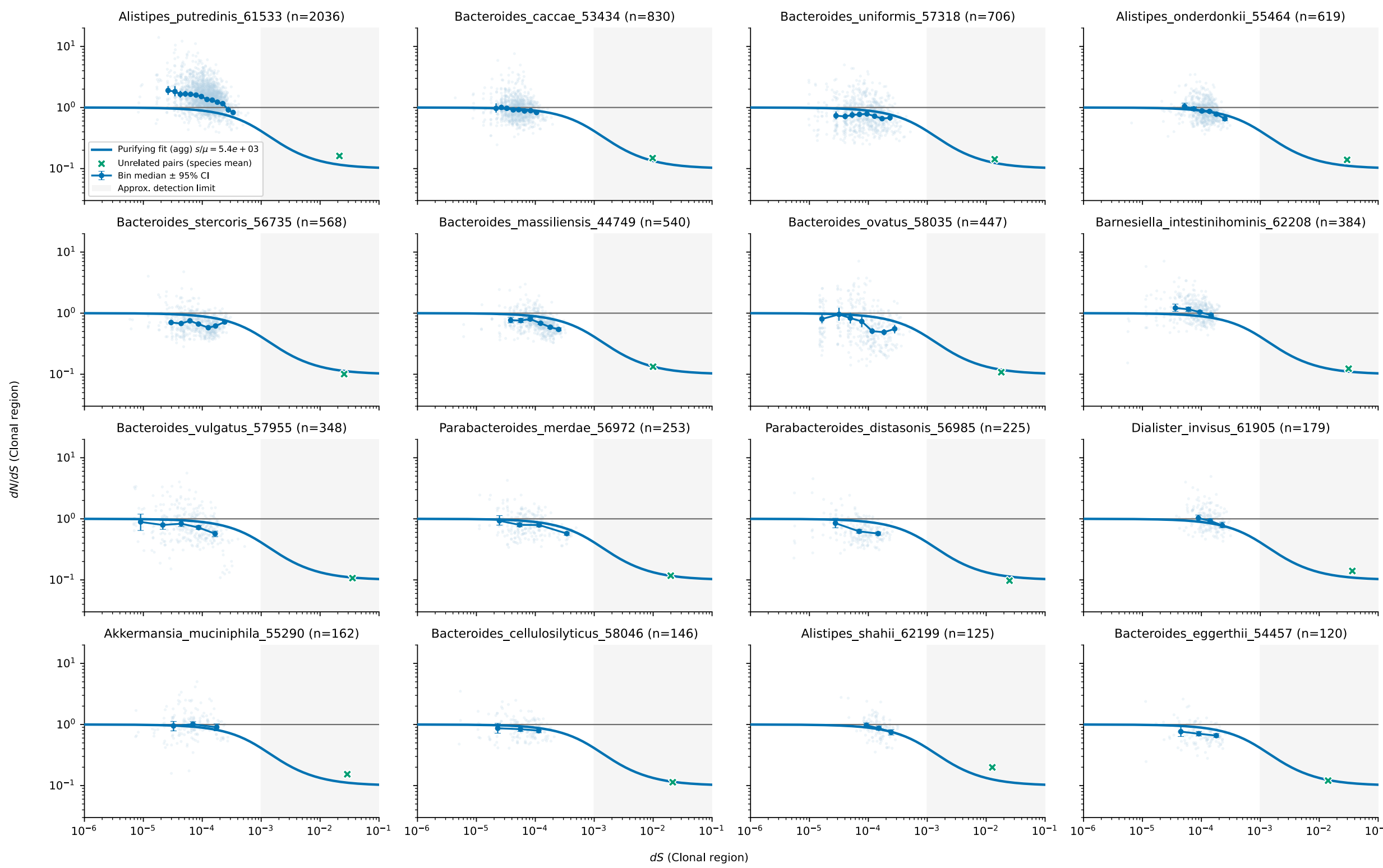

Fig O. An analogous version of Fig E in S1 Text computed for missense mutations only. The blue curve shows the aggregate two-class missense fit from Fig 4 (left), held fixed across species. The data and code needed to generate this figure can be found in `https://doi.org/10.5281/zenodo.22837797`.

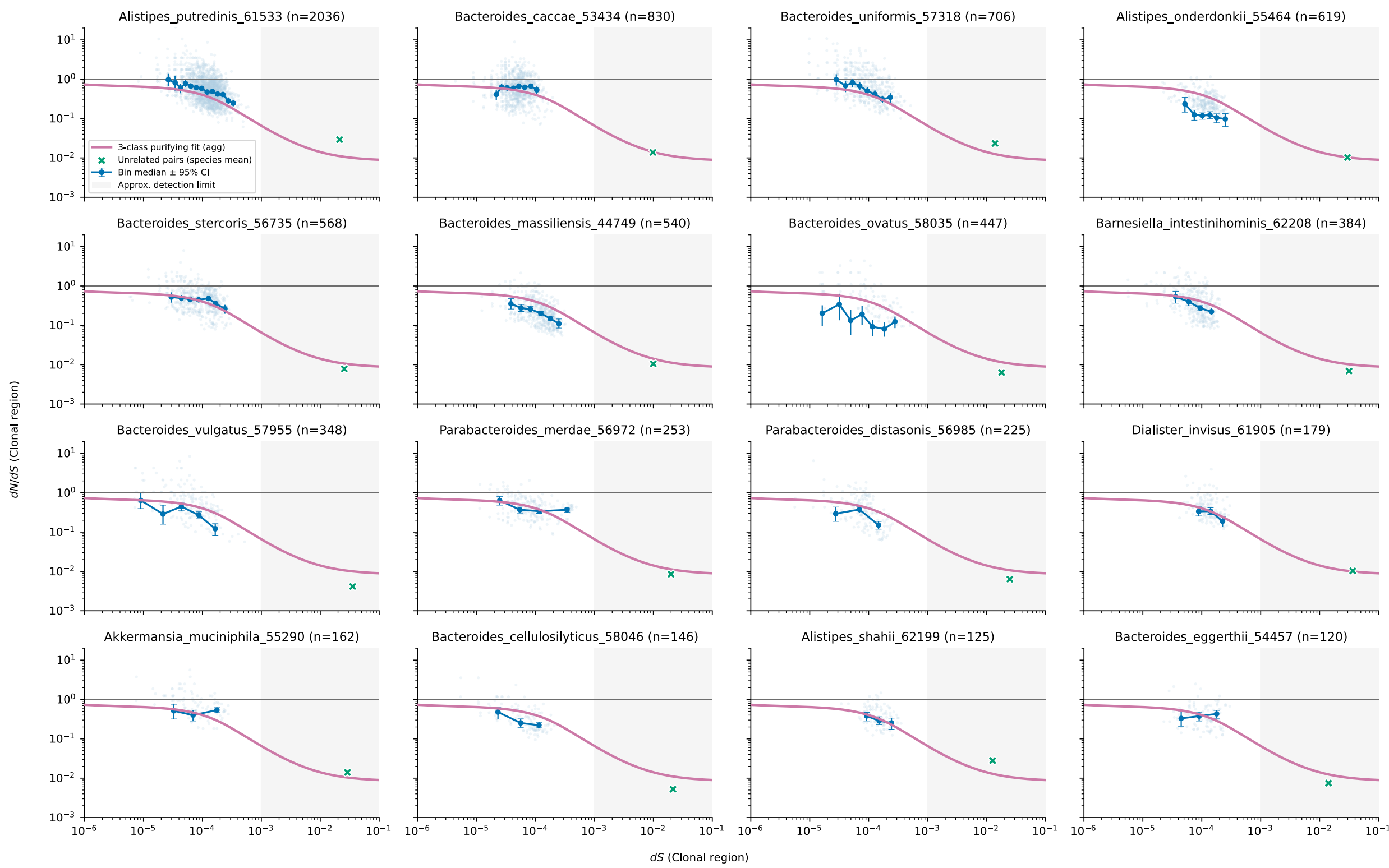

Fig P. An analogous version of Fig E in S1 Text computed for nonsense mutations only. The purple curve shows the aggregate three-class nonsense fit from Fig 4 (right), held fixed across species. The data and code needed to generate this figure can be found in `https://doi.org/10.5281/zenodo.22837797`.

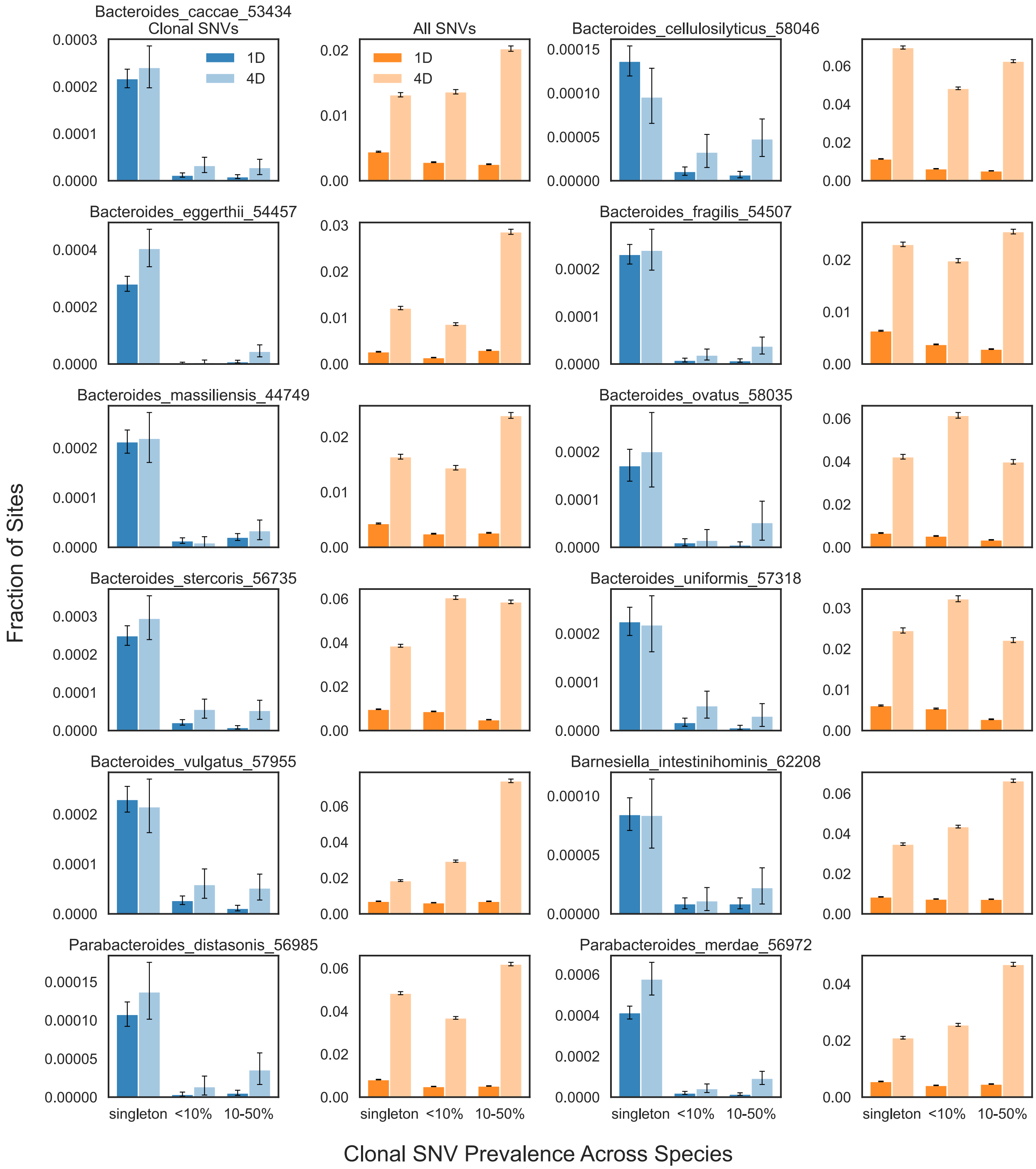

Fig Q. An analogous version of Fig 5 for other commensal gut species. Left panels showing clonal SNVs (1D sites, dark blue; 4D sites, light blue) and right panels showing all SNVs for each species. "Singleton" denotes variants observed in only one host genome within each species. Error bars are 95% Poisson confidence intervals. The data and code needed to generate this figure can be found in `https://doi.org/10.5281/zenodo.22837797`.

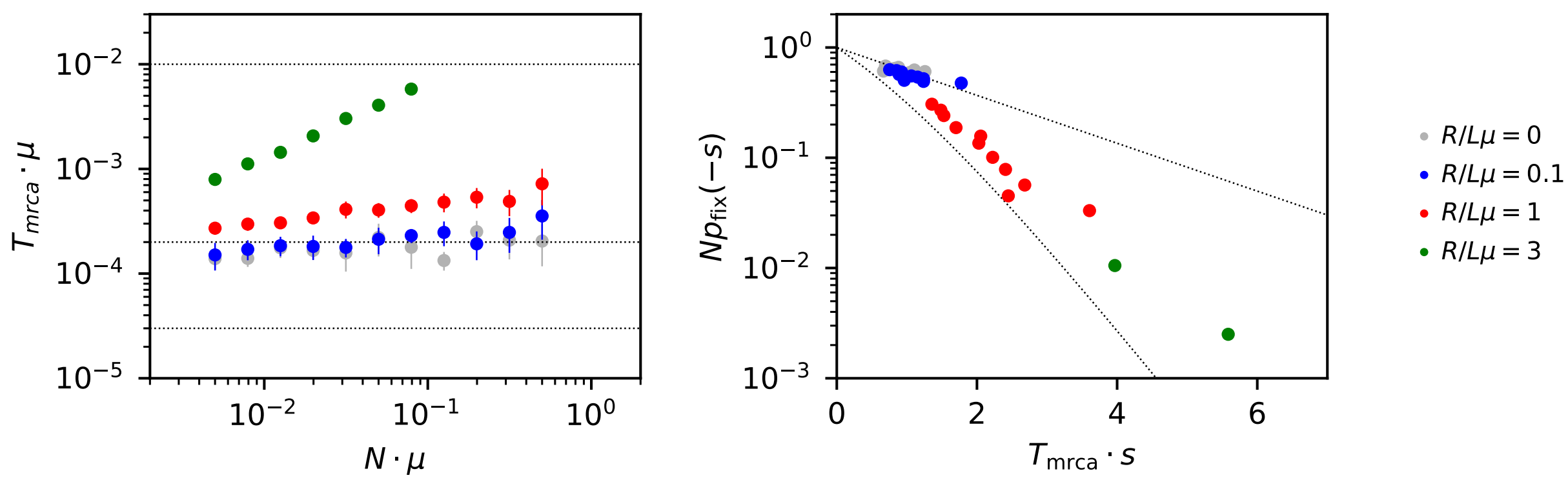

Fig R. Genome-wide simulations of the purifying selection null model generate strong selective interference. Whole-genome, individual-based simulations of the purifying selection model in Fig 3 were performed in two simple metapopulation models, corresponding to the limits of pure intra-host or inter-host competition (Methods Section 5). Using standard diffusion scalings, both models can be depicted with the same set of simulation points, but with different interpretations of the underlying parameters. (a) Realized values of T_{mrca} (estimated from the pairwise diversity at linked neutral sites) as a function of the effective population size N. In the pure intra-host competition model, T_{mrca} refers to the local coalescence time within a single person's microbiome, and N is the corresponding intra-host population size. In the pure inter-host competition model, T_{mrca} refers to the global coalescence timescale ($T_{\text{mrca}} \equiv \overline{d_S}/2\mu$), and N corresponds to the global effective population size (see Methods Section 5). Points show simulation results for four different recombination rates R; in the pure intra-host competition model, we only simulated intra-host transfers ($R \approx R_{\text{int}}$), while the inter-host model only considers transfers between strains in different hosts ($R \approx R_{\text{ext}}$). Dashed lines show three different timescales for comparison: the observed between-host divergence (top), the observed timescale of the clonal dN/dS decay (μ/s_1) (middle), and the lifetime of a single human host (bottom). These data show that the weak fitness costs in Fig 3 generate strong selective interference, with $T_{\text{mrca}} \ll N$. (b) The measured rates of fixation of deleterious mutations (scaled by the underlying mutation rate) for each of the populations in panel a, as a function of the scaled selection strength $T_{\text{mrca}} \cdot s$. Upper dashed line shows the asexual limit $Np_{\text{fix}} \approx \exp(-T_{\text{mrca}}s/2)$ derived in Refs. (55, 57), while bottom dashed line shows an effective single-locus model (75) with $N_e \approx T_c$. These data show that the weak fitness costs in Fig 3 generate strong selective interference (30, 55), which reduces the efficiency of natural selection ($T_{\text{mrca}} \cdot s$) and allows deleterious variants to fix. The data and code needed to generate this figure can be found in `https://doi.org/10.5281/zenodo.22837797`.
