# Supplementary material for: Recombination shapes the dynamics of selective constraint in global populations of human gut bacteria
Source: PLoS Biol. 2026 Oct 12;24(10):e3004052. doi: 10.1371/journal.pbio.3004052 (PMC13690274; doi:10.1371/journal.pbio.3004052)
Supplement: Supplementary file 2 [file pbio.3004052.s004.pdf]

Note: all line numbers, reference numbers, and figure labels refer to the track changes version of the manuscript.

Editor's summary:

Reviewer #1 calls this "a welcome rigorous approach," but has a set of concerns regarding the methodological robustness of the bioinformatics which will need to be addressed, plus some statistical questions and presentational suggestions. Reviewer #2 says that they are enthusiastic about the study, but have a few requests for clarification. Reviewer #3 is also positive but has a number of clarifications and suggestions.

We thank the Reviewers for their positive comments on the manuscript. We have made various changes to address their suggestions, which we describe in more detail below.

Reviewer #1:

This interesting manuscript deals with a long-term controversy in molecular evolution/population genetics: the causes of the variation in relative substitution rates of non-synonymous (dN) and synonymous (dS) substitutions. This ratio (dN/dS) is taken as a proxy for the selection regime, dN/dS being in general less than 1, meaning dominance of purifying selection. About two decades ago, it was shown that dN/dS varied with time since the most recent common ancestor (tMRCA), which was suggested to be caused by purifying selection on weakly deleterious mutations. For recent mutations dN/dS is close to 1 (the difference being caused for example by lethal mutations), whereas for distant bacteria dN/dS tends to low values. A couple of years later it was shown that recombination tracts have lower dN/dS which was interpreted as a consequence of higher local tMRCA on the same effect. Meanwhile other hypotheses were published, including the idea that reversion of non-synonymous mutations could explain these trends. This paper presents a welcome rigorous approach to integrate all this in the analysis of bacteria from the gut microbiota. The key results are that recombination vastly accelerates the decrease of dN/dS with time and the mathematical approach provides estimates of key parameters of the population genetics models that underlie the two hypotheses listed above (and this is an important contribution on itself). The paper is thus timely and very relevant. The text is well written and the figures are clear. I have one major technical issue to raise and some minor comments.

We thank the Reviewer for these positive comments on the manuscript. We have made various changes to address their remaining concerns, which we describe in more detail below.

(1) My main concerns are about the bioinformatics analysis. The authors map reads on genomes and use these mappings to identify recombination tracts and compute dN and dS (and the ratio). That poses two types of problems:

- Mapping. Mapping of metagenomics reads is intrinsically error prone, when compared to using assemblies of complete genomes. This is because the latter are usually from pure

cultures, are sequenced at more uniform depths, and at high coverage. Metagenomic mapping often leads to inaccuracies inflating the number of changes, which poses a more important problem for comparisons of very closely related genomes (than for the distantly related ones). As a result, this may spuriously inflate the effect observed in this work (low contribution of clonal dN/dS because when polymorphism is rare a lot of it is actually sequencing error)

- Alignment. Usually dN/dS is computed on protein-guided DNA alignments. Not sure that was the case here. That would be best, because from my experience standard DNA alignments, especially in the presence of sequencing errors including frameshifts, inflates dN.

While I don't think the following issues change qualitatively the conclusions, they may change quantitatively the results. And the quantitative aspect is an important part (and contribution) of this work. For example, the strange result for one species if dN/dS =2 does suggest problems in the way reads are assigned to genomes. I suggest the authors take one species from the gut that has enough publicly available complete genomes and compare the two approaches (using metagenomes and genome assemblies). The analysis with Staphylococcus is not satisfactory in this respect because recombination was not assessed as in the other analysis, it's a low recombination bacteria, and is not from the gut (so one cannot compare the genome and metagenomic-based approaches). Using complete genomes would also allow to cover more homogeneously the dS range in the figures (which are very low in the dataset).

We thank the reviewer for this comment. We have performed the suggested isolate analysis below. However, before describing this new analysis, we also wanted to clarify two points about our existing mapping and alignment approach:

1.1 Mapping and genotyping. We apologize that our description of the genotyping step was not sufficiently clear in the previous version of the manuscript — we should have better explained that we do not treat individual metagenomic reads as genotypes. Instead, following the previously published quasi-phasing (QP) framework of Garud, Good et al. (2019) [Ref. 28], each sample contributes a single within-host consensus haplotype: an allele is called only where it forms a strong majority (≥80%) of the reads covering that site in that host, and sites at intermediate within-host frequency are masked. Each clonal SNV is therefore a consensus supported by many reads, not a single divergent read, so per-read sequencing and mapping errors are largely averaged out rather than counted as true differences.

The QP framework only considers samples in which the dominant lineage can be genotyped with high confidence, so that low-coverage or ambiguous sites are treated as missing rather than called. This pipeline was subjected to several different validation analyses in Ref. 28, which collectively suggested that the false positive rate is <10% (e.g. <0.1 false positive SNV in a genome-wide comparison of identical strains). These safeguards make it unlikely that the elevated clonal dN/dS in our data is an artifact of accumulated sequencing error. That said, we completely agree with the reviewer that the most direct test is a comparison against gold-standard, pure-culture isolate genomes, and we have now carried out such an analysis below.

1.2 Alignment and annotation. We thank the reviewer for raising this issue, and we apologize for not making the alignment and annotation procedure explicit. To clarify, we do not perform de novo pairwise DNA (or protein-guided) alignments between genomes. Instead, every sample — whether a metagenomic QP genotype or an isolate assembly in the new analysis — is mapped or aligned to a common reference genome, so that all genotypes share a single fixed coordinate system and reading frame. Each coding site is assigned to its reference codon position and classified once as synonymous or nonsynonymous from the reference reading frame (with the reference polarized to the panel's major allele at variable sites). dN/dS is then computed from these fixed per-site classifications rather than from any pairwise genome alignment. Because the codon frame is set by the reference and never re-derived from the data, there is no re-alignment step in which sequencing errors or indels elsewhere in the gene could shift the frame and inflate dN; insertions/deletions relative to the reference are simply treated as missing data at those positions (and, for isolates, flanking sites around frameshifting indels are additionally masked). We have clarified this in the Methods.

1.3 Validation on isolate genomes. Having clarified our metagenomic pipeline, we now turn to the reviewer's suggestion to validate our analysis using isolate genomes from one of the gut species in Fig. 2. To do so, we focused on *Phocaeicola vulgatus* (formerly *Bacteroides vulgatus*), a recombining gut commensal with abundant public isolates. We downloaded 369 high-quality cultured-isolate assemblies from NCBI, built SNV catalogs against the same MIDAS reference used for the metagenomes, and applied exactly the same recombination-detection (CP-HMM) and dN/dS-stratification pipeline that we used for the metagenomic cohort in the main text. (We also downloaded a much smaller set of 41 Alistipes putredinis isolates specifically to investigate the dN/dS ≈ 2 case discussed below). This *P. vulgatus* example should directly address the Reviewer's concerns about our existing *S. aureus* isolate analysis: *P. vulgatus* is a gut species with pervasive recombination, and recombination is now inferred identically across the two data types, so the metagenome- and assembly-based approaches can be compared on equal footing.

Our analysis of this isolate cohort recovers the same qualitative dN/dS separation as in the metagenomic data in Fig. 2: dN/dS remains close to 1 in clonal regions and is substantially reduced in recombined regions and across the full core genome (**Supplemental Fig. B** in the revised manuscript, reproduced in **Fig R1.1** below). This confirms that this signal is not an artifact of metagenomic read mapping, since we also see it in pure-culture assemblies that involve no such mapping.

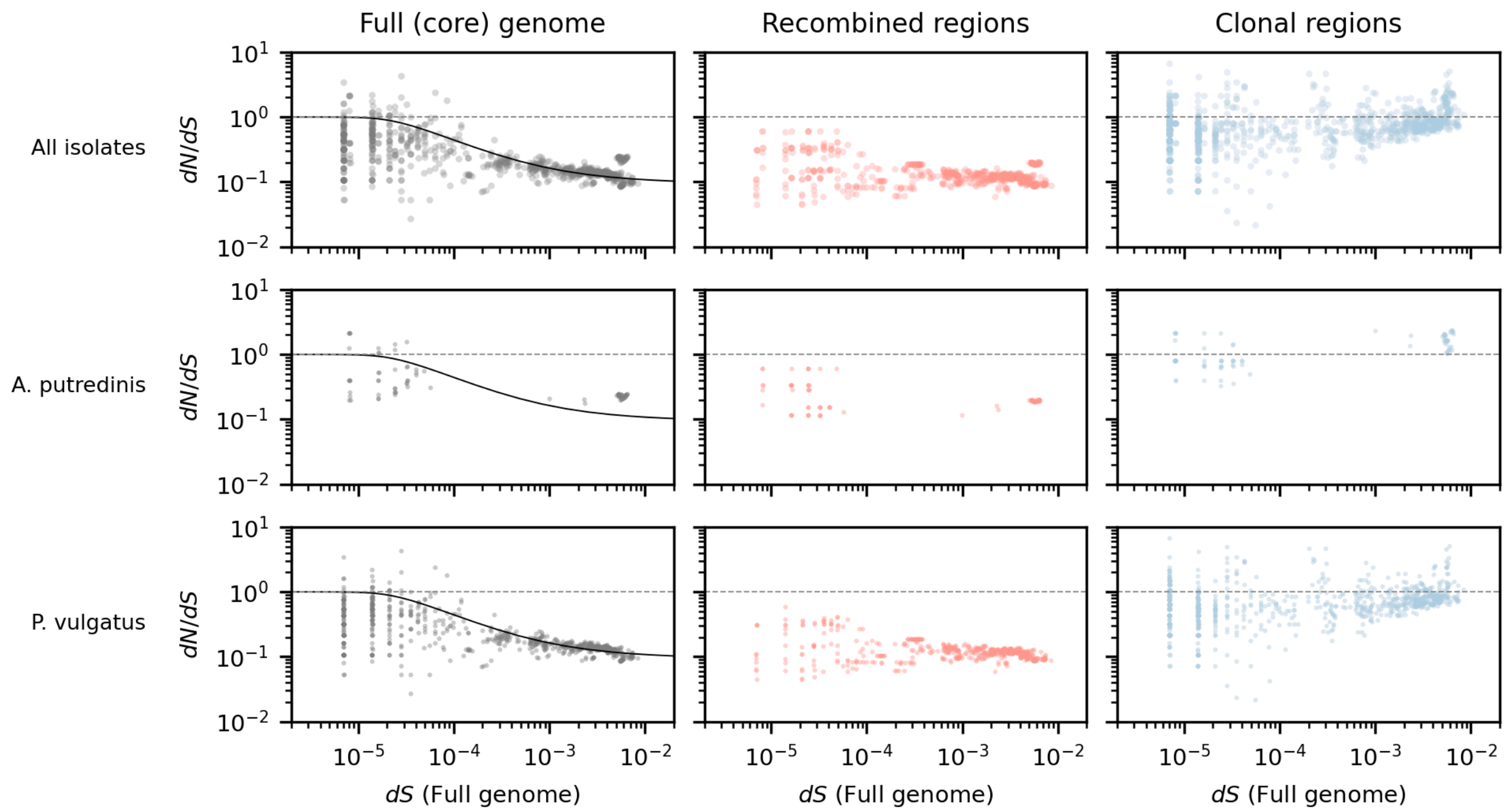

Figure R1.1 (Supplemental Fig B in the revised manuscript). dN/dS dynamics estimated from cultured isolate genomes (41 *A. putredinis*, 369 *P. vulgatus*), with recombination detected per pair by the same CP-HMM. The isolates exhibit the same stratification as the metagenome cohort, confirming the effect is not an artifact of metagenomic mapping.

For *P. vulgatus*, we also performed a direct, head-to-head comparison of the two cohorts on the same plot (**Supplemental Fig C in the revised manuscript, reproduced in Fig R1.2 below**). The stratified dN/dS trends agree and the per-pair data points from the two cohorts overlap the same range of divergence and dN/dS. The clonal-region dN/dS values are also broadly consistent between the two cohorts, both overall and within the missense and nonsense subsets separately, and remain comparable to the pooled purifying-selection fit. This indicates quantitative agreement to within the order-of-magnitude resolution permitted by the intrinsically narrow range of clonal dS.

We emphasize that these low clonal dS values are intentional and central to our method (rather than a limitation of our metagenomic data). It is precisely in this low-divergence regime that individual recombination events remain resolvable against the clonal background, which is what makes the stratification possible; the isolate cohort falls in the same range (dS $\ll 10^{-2}$; **Fig. R1.2**). Extending to more divergent genomes would not add resolution for this particular question, since individual transfer events can no longer be distinguished.

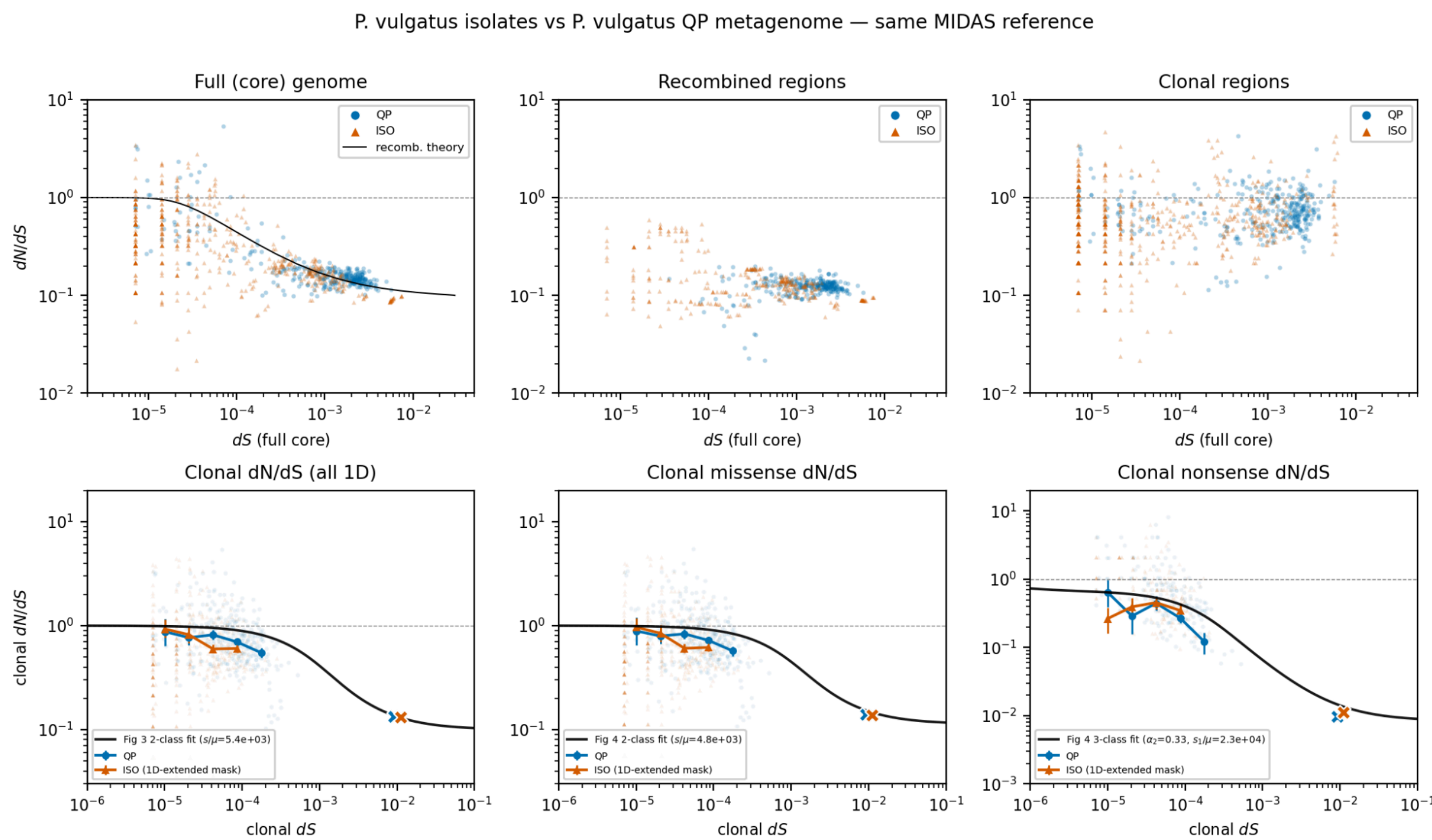

Figure R1.2 (Supplemental Fig C in the revised manuscript). Direct comparison of *P. vulgatus* cultured isolates (ISO) and the quasi-phased metagenome cohort (QP) against the same reference. The stratified dN/dS trends and clonal-region dN/dS broadly overlap between the two. The limited number of pairs per species makes precise parameter estimation difficult, but both approaches are consistent in order of magnitude with the pooled purifying-selection fit (black lines).

1.4 Source of the higher dN/dS values in *A. putredinis.* Repeating this same analysis on the collection of *A. putredinis* also shed light on the source of the puzzling dN/dS=2 result in that species. After further examination, we now believe that this reflects genuine biology rather than a read-assignment artifact. Specifically, manual examination of the SNVs revealed that the elevated clonal dN/dS in this species driven by localized clusters of nonsynonymous (1D) substitutions flanking the detected recombination events that our original HMM method had missed (since it only used information from 4D sites) (**Fig R3.1**). We manually verified that these differences are not explained by paralogy, cross-species mismapping, or copy-number variation. Rather, they appear to mark real recombination at specific loci — most prominently a natural-competence gene — which we believe points to a genuine and potentially rich biological phenomenon that would be interesting to study in future work. We give a fuller account of this result in our response to Reviewer 3's related comment #9 below.

Summary*.* Taken together, we hope these changes have addressed the reviewer's concern about the bioinformatics analysis. We have made our full pipeline available, covering the download of all qualifying isolate genomes from NCBI, construction of the SNV catalog, and recombination analysis. We hope this will allow the field to test these patterns more broadly across other well-sequenced species.

(2) The authors work suggest that dN/dS decrease would be strongly dependent on species recombination rates. This is surely outside of the scope of this paper, but it would be relevant in the future to test this, i.e. pick bacterial species with increasing recombination rates and assess the shapes of decrease in dN/dS.

Yes, we agree – there are some hints at this already in our response to Comment #2 from Reviewer 3 below. However, as we discuss there, these differences are not only driven by the average recombination rate of a species, but also by the whole joint distribution of recombination rates and coalescence times. We are excited to investigate this more systematically in future work, which we suspect will likely require larger sample sizes to tease apart these different factors.

(3) There are some statistical biases in the analysis for which I have no great solution, but should be mentioned/discussed. i) Strictly speaking dS and dN/dS are not statistically independent. This may pose a problem in that random fluctuations in dS in the clonal descent lead to extreme positive values resulting in lower dN/dS.

Thank you for flagging this – we had previously employed a Poisson thinning approach to correct for precisely this issue (lines 438-446 in the Methods), but this comment made us realize that we hadn't mentioned this fact anywhere in the main text. We have therefore revised the figure captions of Figs 2 and 3 to point out that different dS estimates are used for the x and y axes.

(4) ii) The identification of recombination tracts requires high density of polymorphism. Hence, highly divergent tracts are more likely to be identified than exchanges between very similar sequences. This means two things: the cumulative effect of recombination may be slightly underestimated (because the missing events carry little signal), and the individual effect of recombination events is over-estimated (because the events that are accounted for are the ones with highest levels of polymorphism).

Yes, we agree – in reality, there will be a continuum of recombination rates that import fragments with different divergence levels. Our current study is only considering the rates and contributions above a critical divergence threshold that can be reliably detected with our HMM approach, but we are almost certainly missing recombination events from more closely related strains. Thus, our "clonal" dN and dS estimates could still contain some residual contributions from these undetected recombination events. This could potentially even account for some of the high frequency mutations in the top right corner of Fig. 5A. There is not quite enough data to say much more about these events in our current manuscript, but we have added an acknowledgement of this limitation in the Discussion (lines 382-387) and Methods Section 5 (lines 654 and 696-703).

(5) I suggest adding the methods in the main text, instead of leaving them as supplementary material. That's the (good) standard of PLoS Biology.

Thank you for this suggestion. We have implemented it in the revised version of the manuscript. (We will defer to the editors for guidance on the final version, should the manuscript be accepted for publication.)

(6) Very minor. In figure 1A I would suggest to have as short term only the adaptive and put a middle-term for neutral. Neutral would be the reference.

Thank you for this suggestion. After considering it carefully, we would still prefer to list both adaptive and neutral under the short-term, since we feel it is the more conservative choice given the data we've presented (which don't directly demonstrate adaptation). As a compromise, we've changed "neutral" to "effectively neutral" to better indicate that we aren't making a claim about true neutrality.

(7) I suggest adding explicitly also the git repertoire for the program to identify recombination using an HMM. It would be useful to the community.

Thank you for this suggestion. We've added a link to the corresponding Github repository for this tool.

Reviewer #2:
[identify themselves as Nandita Garud and Peter Laurin]

Summary

This paper makes an important contribution to the study of bacterial evolution by showing that the decay of dN / dS can be highly confounded in recombinant bacterial populations. Thus, the time in which we can observe evolution directly acting between clonal descendants may be very short, and the range of the curve informative to evolutionary models may be quite small. Nonetheless, the authors show convincingly that a purifying selection model may be a decisively better fit to the dN / dS curve of species in the human gut microbiome than a recently-proposed model of adaptive reversion. Overall, their findings have broad implications to the interpretation of dN / dS in bacteria and limitations thereof, and provide further insight into the evolution of gut commensals.

We overall are very enthusiastic about this work, and have minor comments/questions for the authors.

We thank the Reviewers for these positive comments on the manuscript. We have made various changes to address their remaining concerns, which we describe in more detail below.

1. Can you speculate why the "effective" fitness costs estimated by the decay in dN/dS might differ from those estimated in other methods (e.g. LD, SFS)

This is a good question. We don't have a definitive answer at the moment, but we suspect that this discrepancy is due to the fact that these other methods are imposing their own set of assumptions (e.g. complete linkage equilibrium, little genetic draft) which either (1) don't hold in practice or (2) break down for the subset of closely related strains we are considering here. We agree that it would be interesting for future work to try to reconcile these differences, perhaps using variants of the forward time models described in our response to comment #1 from Reviewer 3 below. We are also exploring variants of this model with spatiotemporally varying selection pressures, which could potentially unite the two pictures above by producing systematically different fitness costs on short vs long timescales.

2. Given there is such a small amount of curve to fit selection coefficients, it seems that the equilibrium value of dN / dS (i.e. in well-recombined strains) may have a large effect on the estimation. How sensitive is the fit of s/mu to the equilibrium value?

This is also a good question, and it prompted us to test the issue directly in a new supplementary figure (**Supplemental Fig H** in the revised manuscript, reproduced in **Fig R2.1** below). We'd first like to clarify how the equilibrium dN/dS enters our fit: at present, it is used only to set the neutral fraction α_0 (the long-term asymptote of the two-class model), while the synonymous divergence dS at which this equilibrium is reached does not enter the fit at all. To probe sensitivity to both of these quantities, we held the clonal dN/dS points fixed and re-fit s/μ while scanning a grid of artificial equilibrium values: first varying α_0 alone (**Supp. Fig H,** panel B), and then also inserting the equilibrium value as an explicit anchor in the weighted least-squares at its (dS, dN/dS) location (panel C). The dashed box marks the full range of equilibrium dN/dS and dS values that we actually observe across the 43 species (panel A), and the star marks the pooled values used in the main text.

Reassuringly, across this empirically observed range the inferred s/μ shifts by less than ~20%. We note, however, that the robustness here reflects the stability of the pooled estimate to the assumed equilibrium value, and is not a claim that s/μ is tightly or uniformly determined. If we instead fit each species naively from its own (much sparser) clonal data, the estimates span roughly 10^3–2×10^4, which reflects the sensitivity to the shape of the initial departure from neutrality (dN/dS ≈ 1). We feel this reinforces the broader caveat we raise at the end of the Discussion: because recombination erases clonal segments so quickly, the intermediate regime where dN/dS transitions from neutral toward its equilibrium is effectively unobservable, so the real value of this analysis lies in placing order-of-magnitude constraints on the underlying distribution of fitness effects rather than in pinning down a single precise selection coefficient.

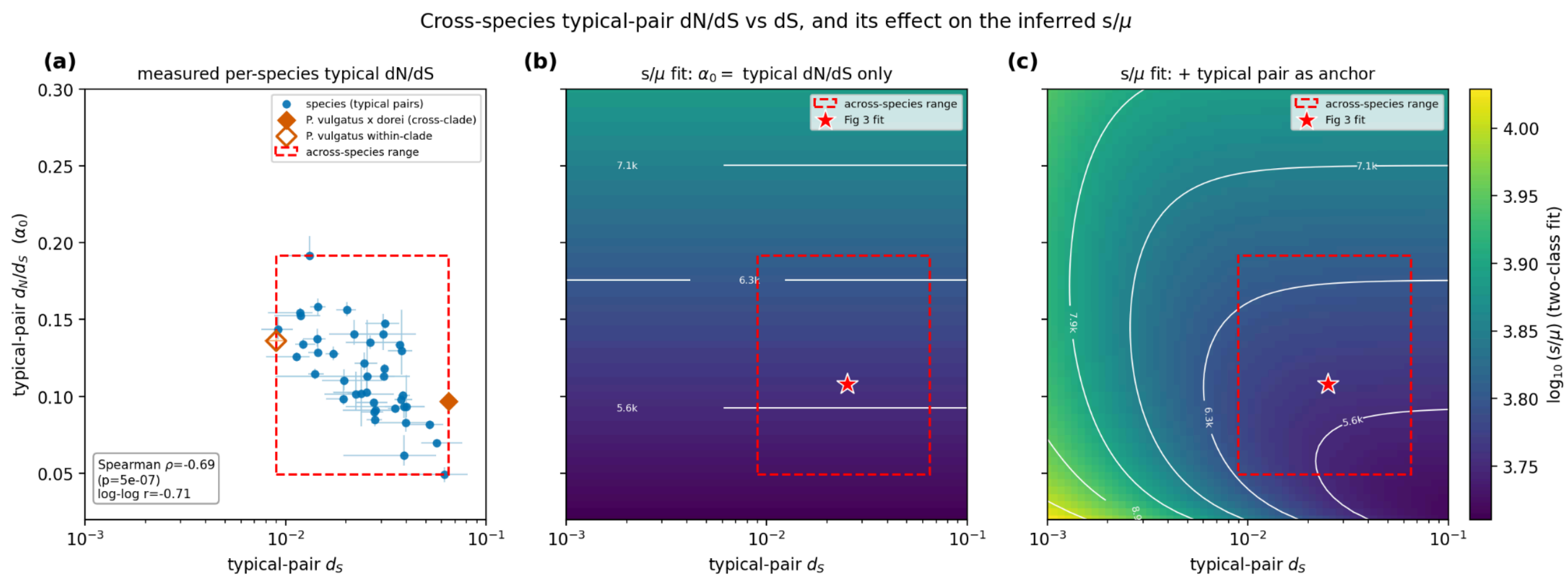

Figure R2.1 (Supplemental Fig H in the revised manuscript). Cross-species variation in typical-pair dN/dS has a negligible effect on the inferred strength of selection. (a) Measured typical-pair dN/dS versus synonymous divergence dS, one point per species (the green crosses of Fig. 3); the dashed box marks the full across-species range. (b,c) Refitted clonal s_1/μ as the assumed typical-pair dN/dS and dS are swept over the same plane (red star: operating point of Fig. 3), without (b) and with (c) the typical pair as a fit anchor. Across the entire box s_1/μ stays within ~5–8×10³.

3. Could the authors make clearer what TMRCA is in this paper? Is it the coalescence time of the entire species (e.g. Fig 1A) or the average coalescence time between two genomes? Are they meant to be the same given extensive recombination across strains of the same species? Could the authors provide a little more explanation? Maybe this is well defined in Liu and Good 2024 PloS Biology and requires a small citation here.

We apologize for the ambiguity. Here we are using TMRCA to refer to the average pairwise coalescence time within the species (which, as the Reviewers anticipated, is also close to the genome-wide TMRCA between any two genomes given the amount of recombination in many of these species). We have edited the text (line 91) and Fig. 1A to more clearly indicate this definition.

4. Line 161: "Fig. 2D shows that the dN/dS levels on the clonal backbone remain close to one even as the nominal dS values span more than three orders of magnitude (10^-5 < dS < 10^-2; Fig. 2D). However, because these genome-wide dS levels are inflated by recombination, they overestimate the true divergence time between closely related strains."
This is a very important point, and critical to understanding Fig 2. The initial lack of decay when plotted by dS confused us on first reading, as well. We recommend adding this context earlier to the Fig 2 section: Quantifying the impact of homologous recombination on dN/dS dynamics because otherwise the intuition that there should be a decay with dS doesn't line up with the data.

Thanks for this suggestion – we have edited the legend of Fig. 2 to explicitly point this out.

5. How were the parameters chosen for the "recombination theory" line in Fig 2B? Is it by visual inspection/fitting from fig S1? If so, then in what sense are there no free parameters with the phenomenological model? But the point is well taken that the dynamics of dN/dS can be described well with the characteristics of recombined fragments alone.

We apologize for the lack of clarity here (Reviewer 3 noted a similar issue in their comment #2 below). The Reviewers are correct that our phenomenological model has an important free parameter: the dependence of the recombined fraction of the genome on dSc. Our point is that once this curve is determined (which can be done using synonymous sites alone), there are no *additional* free parameters necessary to reproduce the dN/dS curve (beyond the two average levels in Figs. 2C and D). We have edited the text on lines 134-136 and in Methods Section 3 to more explicitly point this out.

Furthermore, as part of our response to comment #8 from Reviewer 3 below, we have replaced the previous “by eye” fit of the hill function in Fig. S1 (now **Supplementary Fig A**) with a more rigorous nonparametric approach (Methods Section 3), which should make it more clear how we actually do the fit.

Signed
Nandita Garud and Peter Laurin

Reviewer #3:

[Identifies himself as Daniel Weissman]

In this manuscript, Liu and Good use data from many gut microbiome species and Staphylococcus aureus to show that the observed decay of pairwise dN/dS with genetic distance within species is primarily driven by recombination. I think this is a fascinating result that suggests a way forward for a controversial question. Fig 2 is a striking demonstration of the importance of recombination, and Fig 3 clearly illustrates the true, slower selective dynamics in non-recombined regions of the genome. I learned a lot from the manuscript.

We thank the reviewer for these positive comments on the manuscript. We have made various changes to address their additional questions and concerns below:

Major question:

(1) The one big thing that I think could be clarified is the extent to which the authors have found a full, self-consistent model for the data. A key motivation for this work is Torrillo and Lieberman 2024, which pointed out that the pervasive modest purifying selection inferred from previous dN/dS analyses could lead to Muller's ratchet. In other words, the simple model that described a single locus might break down when applied simultaneously throughout the genome. With the authors' inferred selective coefficients, how frequent would selection have to be and how big would the population need to be to avoid Muller's ratchet? Or am I just totally missing something? 321-331 in the Discussion kind of gets at this, but it's more focused on how to

derive the effective model from micro dynamics, whereas I'm curious about the consistency of the effective model itself.

Thanks for raising this issue. This is also something we're very curious about, but we feel that it is intimately bound up with this larger question about the connection with the underlying microscopic dynamics:

It's worth emphasizing that Torrillo and Lieberman (2024) considered a rather extreme microscopic model where there was effectively no inter-host competition. Once a deleterious mutation was able to fix within one of their hosts, it could only be removed by a subsequent reversion. In hindsight, it is clear that such a model will always have trouble attaining a low dN/dS value as soon as there is any appreciable chance of hitchhiking within hosts (whether through Muller's ratchet or other mechanisms).

A more interesting question (which we assume is closer to what you're asking above) is whether a more realistic model of inter-host competition can still efficiently purge mutations on longer evolutionary timescales (e.g. ~T_mrca) even when they are able to neutrally hitchhike within individual hosts. Here too, we believe that the answer depends a lot on what assumptions one makes about the underlying microscopic dynamics.

One can consider the simplest possible model (the opposite extreme of one employed by Torillo & Lieberman) where each host is represented by a single strain, which compete and recombine with each other as part of a well-mixed global population. As far as we are aware, even this simple model of background selection not yet understood theoretically, since the "chunk-like" nature of bacterial recombination is sufficiently different from both the asexual and linear chromosome cases that have been analyzed previously (e.g. Good et al PLoS Genet). In the revised manuscript, we have performed a new set of individual-based simulations of this model (**Supplemental Fig R** reproduced in **Fig. R3.1** below) to show that there is also strong linked selection in this case, depending on what one assumes about the underlying rates of recombination. (We would argue that Muller's ratchet per se is not the issue here, but rather the shortening of the global coalescence timescale and the reduction of the efficiency of selection.) Interestingly, even the simulations of this model are surprisingly difficult to perform for the range of parameters that are necessary; even with the most aggressive diffusion scaling, we were not able to reach the upper end of the plausible effective population sizes for these bacteria.

However, there are some important caveats that make us hesitant to claim that this model is truly inconsistent with the data. One issue is that the behavior of the model is very dependent on the underlying rate of recombination. We do not know this parameter directly, but tend to constrain it from the realized rate of successful transfers. In the presence of widespread linked selection, the link between the underlying and apparent rates is not so clear, and would need to be self-consistently fit to the model (this is related to the Reviewer's Comment #2 below). More generally, the simplest version of the model omits a lot of relevant factors (e.g. spatial structure, epistasis, and fluctuating selection) that could plausibly alter the strength of linked selection, but are still very poorly understood theoretically. For these reasons, we believe that a full answer to

this question will likely need to remain a topic for future work.

However, we appreciate that omitting the above discussion may implicitly suggest that the “simplest model” indeed fits the data. We have therefore revised this paragraph of the Discussion (lines 351-358) to explicitly highlight the issue of selective interference, and have added a longer discussion in the Methods (Section 5). We hope this strikes the right balance between highlighting the need for more theoretical and empirical work on this topic, while remaining appropriately conservative about the possible conclusions.

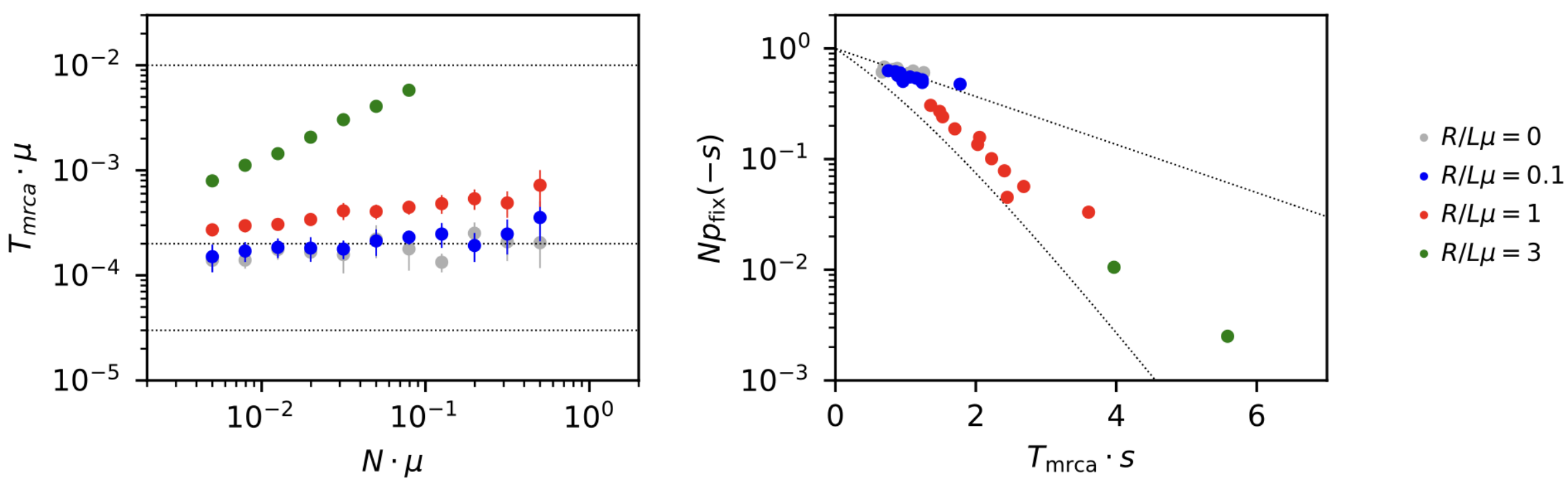

Figure R3.1 (Supplemental Fig R in the revised manuscript). Whole-genome, individual-based simulations of the purifying selection model in Fig. 3 were performed in two simple metapopulation models (Methods Section 5), corresponding to the limits of pure intra-host or inter-host competition, respectively. (a) Realized values of Tc (estimated from the pairwise diversity at linked neutral sites) as a function of the underlying population size N. Points show simulation results for four different underlying recombination rates. Dashed lines show three different timescales for comparison: the observed between-host divergence (top), the timescale of the clonal dN/dS decay (mu/s_1) (middle), and the lifetime of a single human host (bottom). These data show that the weak fitness costs in Fig. 3 generate strong selective interference, with Tc << N. (b) The measured rates of fixation of deleterious mutations (scaled by the underlying mutation rate) for each of the populations in panel a, as a function of the scaled selection strength Tc*s. Upper dashed line shows the asexual limit Npfix = \exp(-Tc s/2), while bottom dashed line shows an effective single-locus model with Ne = T_c. These data show that the weak fitness costs in Fig 3 generate strong selective interference, which reduces the efficiency of natural selection and allows deleterious variants to fix.

Minor questions:

(2) Related to the major comment above, I would add just a sentence or two more emphasizing that the recombination-accumulation function (S5) is just a fit and doesn't come out of any dynamical model, and that it requires _super-linear_ accumulation at short time scales.

Thanks for pointing this out. This turned out to be a more interesting and subtle point than we had anticipated. To start, we agree that the recombination-accumulation function is a purely empirical fit (and doesn’t come out of any dynamical model). However, after treating the noise

more carefully in the revised version of the manuscript, we've found that the relevant empirical curve is *not* the average value of f_r(dS_c), as we had described it in the previous version, but rather the reverse expectation, E[dS_c|f_r] (in other words, averaging the old version of Fig. S1 over the x axis rather than the y axis).

This subtle difference is important because the reverse expectation E[dS_c|f_r] will generally depend on the overall distribution of dS_c values, in addition to the shape of the recombination function p(f_r|dS_c). We suspect that this double averaging is the cause of the apparently "non-linear" recombination accumulation function that the reviewer noted above. That is, the dominant way to get an intermediate dN/dS value is to have a larger coalescence time T that has a typical clonal divergence, but happens to get an anomalously low f_r by chance. Our previous study (Liu & Good PLoS Bio 2024; Ref. 34) found that these anomalous values of f_r could occur even when the expected value of E[f_r|dS_c] was a linear or sub-linear function of dS_c.

A closer examination of the data appears to support this hypothesis. The strain pairs with intermediate dN/dS values are rare and unevenly distributed: only about 2% of pairs in this transition band sit above the long-term dN/dS plateau, and these are concentrated in a few species (for example, about 40% from *Bacteroides caccae*, and about 70% from the top three) in a way not explained by the sample size, since the most heavily sampled species contributes almost none (for example, *Alistipes putredinis*). However, the recombination accumulation patterns in our previous study (Ref. 34) almost perfectly explains this difference: *B. caccae* had an unusual subset of pairs with very few detected recombination events, while *A. putredinis* had a much lower number of downward fluctuations in f_r.

For these reasons, we wouldn't read too much into the super-linear fit in the previous version of Fig. S1. While this might be the correct average to use for predicting dN/dS, we suspect that the dynamics of the true recombination accumulation function are better probed with the traditional average E[f_r|dS_c], since it depends less strongly on the (unmodeled) distribution of dS_c. In our previous study in Ref. 34, this function tended to exhibit linear or sub-linear dependence on dS_c for most species – though with substantial fluctuations that are apparently important for certain quantities like dN/dS.

In the revised manuscript, we have now substantially revised Section 3 of the Methods to explain this new approach for parameterizing the recombination accumulation curve, and we have updated Fig. S1 (now **Supplemental Fig A**) accordingly. As part of this update, we have switched from the parametric Hill-function form to a more systematic non-parametric approach, which should further emphasize that this is a purely empirical fit. We are grateful to the Reviewer for prompting us to look into this issue.

I guess one may get something like that naturally in the model because selection is promoting recombination events that fix accumulated deleterious mutations, but this isn't something that anybody has worked out, is it? Do the authors think this is an interesting area for future work?

We agree that studying the recombination accumulation dynamics in the genome-scale models like comment #1 would be very interesting (since they are essentially unknown to our knowledge). This is one of the things we'd like to investigate in a larger theoretical study using the forward-time simulations above; we discuss this issue briefly in the new Methods Section 5 (lines 650-659 and 731-734).

However, our intuition was that the direction of the effect you alluded to above might correspond to a *sub-linear* (rather than super-linear) dependence on dS_c, since a super-linear dependence would actually have *less* recombination when dS_c is below the inflection point. But this is all very speculative – it would be very interesting to go in and look in simulations.

(3) Is the spread in dN/dS values around the mean way larger than what would be predicted by naively applying the simple purifying selection model? What about within species?

Thanks for raising this point. To address this question, we simulated the expected spread in dN/dS under the phenomenological purifying-selection model, drawing the nonsynonymous counts from a Poisson distribution while preserving each pair's real 4D/1D site densities, clonal-region length, and the identical synonymous thinning used in the figure. Comparing matched dS bins, much of the apparent scatter is consistent with finite-site sampling noise, but a real overdispersion remains: the observed spread is ~1.5-1.8× wider than the model predicts, at a similar level across species (**Supplemental Fig I** in the revised manuscript, reproduced in **Fig R3.1** below). In addition to variation across species, some of this overdispersion might also reflect short recombination events with very few SNVs that were undetected (related to Comment #4 by Reviewer 1 above). We have added the analysis in the revised manuscript (end of Methods Section 2).

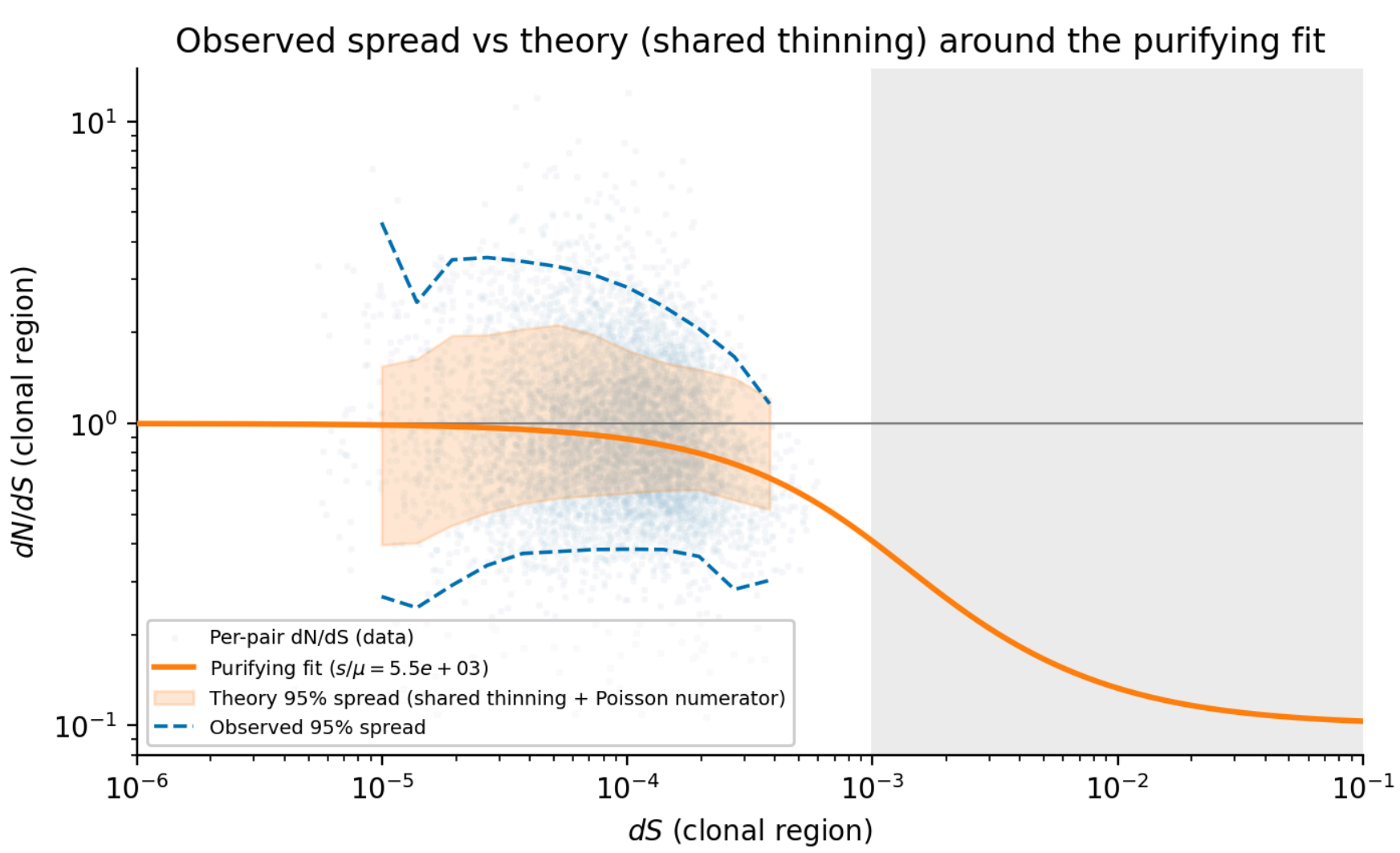

Figure R3.2 (Supplemental Fig I in the revised manuscript). Spread of clonal dN/dS vs. the purifying-selection model. Per-pair dN/dS vs dS (points), the fitted curve (orange), the model-predicted 95% spread from finite-site sampling alone (orange band), and the observed 95% spread (dashed). The observed scatter is ~1.5-2x wider than the Poisson resampling model predicts.

(4) It looks like there's a downward trend in the mean dN/dS values across species, with more diverse species having lower mean dN/dS. Am I fooling myself there? Could that be a trace of truly weakly selected mutations?

Great observation – we examined it directly by computing the mean synonymous divergence (mean dS) and the typical-pair (long-term, fully recombined) dN/dS for each species, with the same Poisson-thinning procedure to remove artificial correlations (**Supplemental Fig H** in the revised manuscript, reproduced in **Fig R3.3** below**)**. We can confirm that there is a significant negative association (Spearman rho = -0.69, p = 5×10^-7, n = 42 species): the mean typical dN/dS declines from approximately 0.15 in the least-diverged species to approximately 0.05 in the most-diverged, across a roughly seven-fold range of mean dS (about 0.009 to 0.06). We note that a similar pattern was reported by Rocha et al. (2006), who found a continued change in dS/dN from ~7 to ~25 (equivalent to a dN/dS from ~0.14 to ~0.04) across their comparisons of bacterial strains within species complexes.

We agree that this pattern could be consistent with a subset of extremely weakly deleterious mutations that continue to be purged on these longer timescales. Heuristically, because 1 - dN/dS approximates the fraction of nonsynonymous mutations purged by divergence dS, the ~10% decline in dN/dS across this window implies that ~10% of nonsynonymous mutations carry costs of order s/mu ~ 10^2. We have now added this result in the revised manuscript (**Supplemental Fig N,** related to the reviewer's comment #5 below).

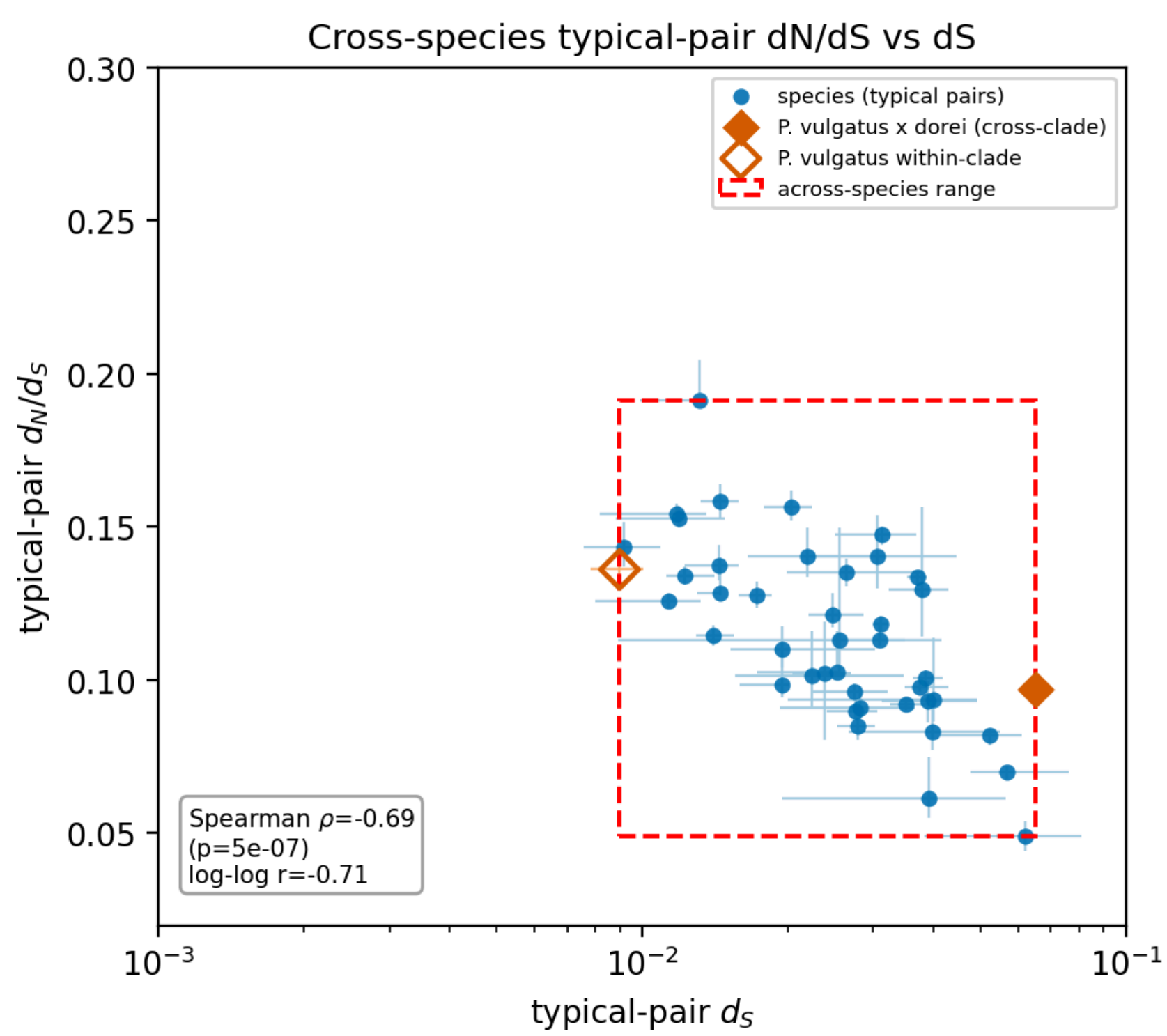

Figure R3.3 (Supplemental Fig H in the revised manuscript). Measured typical-pair dN/dS versus synonymous divergence dS, one point per species (the green crosses of Fig. 3), with bars showing the central 80% inter-pair spread; the dashed box marks the full across-species range. Orange diamonds mark *P. vulgatus* within-clade (open) and cross-clade *vulgatus* vs *dorei* (filled) pairs, a single species spanning a wide divergence range.

(5) Would it be easy to add supplemental plots showing the bounds on the cumulative DFE from (S13-14)?

Thanks for this suggestion. We have added a new figure along these lines (**Supplemental Fig N**).

(6) When talking about the bounds on s, it's a bit tricky to decide when to leave things in terms of other parameters ("s << 10^5 mu") and when to plug in typical values of those parameters to allow writing absolute numbers ("s << 10^-4"). In the first paragraph of the Discussion, I think it might be nice to add a line turning the lower bound s \gtrsim 1/TMRCA into absolute numbers to complement the s << 10^-4 upper bound.

Thanks for this suggestion – we have added the 1/TMRCA conversion to that paragraph.

(7) I found 341-343 hard to understand. I think 954-967 in the supplement explains what the authors are saying there. Perhaps a chunk of that paragraph could be moved up to the Discussion?

Thanks for flagging this. We have now expanded these sentences into a full paragraph by moving the text as suggested.

(8) It would be nice to have a curve showing some kind of simple binned or smoothed average of the data in Fig S1.

Thank you for this suggestion – this turned out to be critical in helping us diagnose the issue in comment #2 above. As described in the response to comment #2 above, we have replaced the previous Hill function fit in Fig. S1 (now **Supplemental Fig A**) with a nonparametric binned average (+ spline fit), which we use as input to our phenomenological model.

(9) I'd invite the authors to feel free to throw in a line of wild speculation about Alistipes putredinis if they want.

Thank you for raising this point. Reviewer 1 also commented on the outlier behavior of Alistipes putredinis, which prompted us to take a more thorough look at the underlying data. Interestingly, we discovered that its anomalously high clonal dN/dS (≈2) is not a mapping artifact, but instead reflects recombination that our detector had missed. Our CP-HMM identifies transfers from the density of synonymous (4D) differences; a tract imported from a donor that differs mainly at nonsynonymous (1D) sites therefore carries little 4D signal, and the HMM truncates it where that

signal fades, leaving a 1D-dense, 4D-poor flank misclassified as clonal. These missed nonsynonymous differences inflate the apparent clonal dN/dS. We confirmed this mechanism in individual worked examples from both the metagenomic and isolate cohorts (**Supplemental Fig F** in the revised manuscript, reproduced in **Fig. R3.4** below).

To address this issue, we updated our CP-HMM package with a new post-processing step that extends the boundaries of detected recombination events outward over adjacent nonsynonymous-rich sequence, reassigning these missed differences to the recombined region. Applying this correction brings the *A. putredinis* clonal dN/dS down from ≈2 to a nearly neutral value of ≈1, in line with the other species, while leaving the pooled selection-strength estimate essentially unchanged (**Supplemental Fig G** in the revised manuscript, reproduced in **Fig. R3.5** below). We have included these new analyses in the Methods and added a corresponding discussion to the main text (lines 189-193, 216, and 384).

We found it intriguing that all three *Alistipes* species in our cohort show appreciable corrections of this kind, whereas the *Bacteroides* species are essentially unaffected (**Supplemental Fig G**). The phenomenon is not unique to *Alistipes* — we also see appreciable adjustments in *Akkermansia muciniphila* and *Dialister invisus* — but why *Alistipes* shows such a strong and consistent trend remains a puzzle to us. One possibility is that it reflects a genuine signal of rapid adaptation: in a preliminary scan of where these nonsynonymous-rich windows fall along the *A. putredinis* genome, the most prominent hotspot coincides with a natural-competence protein (**Fig. R3.6**). Fully understanding this phenomenon, and assessing its prevalence across gut bacteria, is beyond the scope of the present work, but we think it is an interesting direction for probing the functional significance of the pervasive recombination observed in these populations.

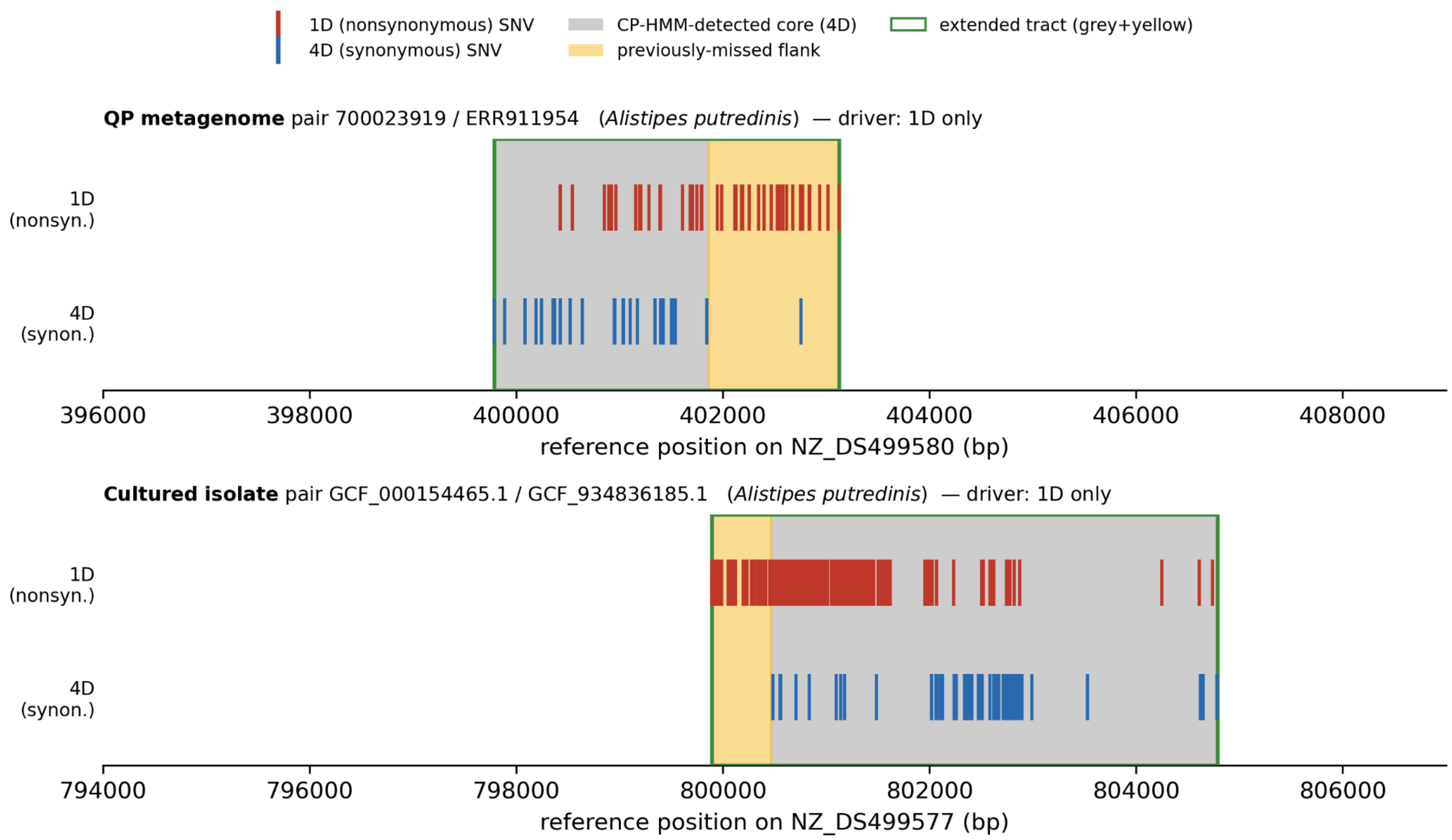

Figure R3.4 (**Supplemental Fig F** in the revised manuscript). Two A. putredinis close pairs (metagenome, top; isolate, bottom) showing pairwise SNVs at nonsynonymous (1D, red) and synonymous (4D, blue) sites. Because the CP-HMM detects transfers from 4D density, it captures the tract core (grey) but truncates over the adjacent 1D-rich, 4D-poor flank (yellow), misclassifying it as clonal; extending the boundary (green) reassigns these stranded nonsynonymous differences to the recombined region.

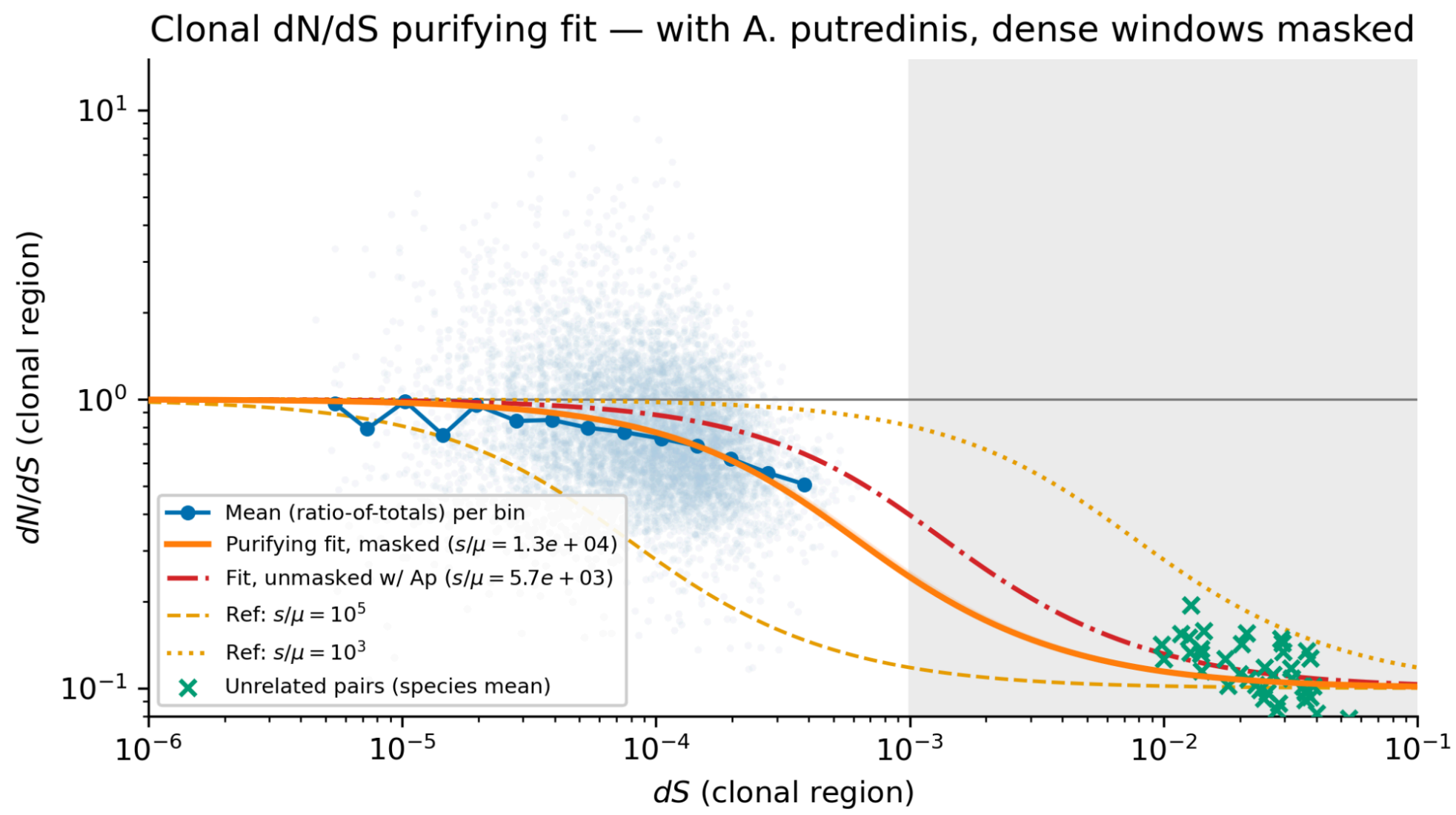

Figure R3.5 (**Supplemental Fig G** in the revised manuscript)**.** Clonal dN/dS fit including *A. putredinis* after extending recombination boundaries over the missed nonsynonymous-rich flanks. The corrected estimate ($s/\mu \approx 1.3\times10^4$) is close to the no-Ap fit ($\approx 1.1\times10^4$) and about

twice the main-text reference curve ($\approx 5\times10^3$), confirming the order of magnitude is robust to the correction.

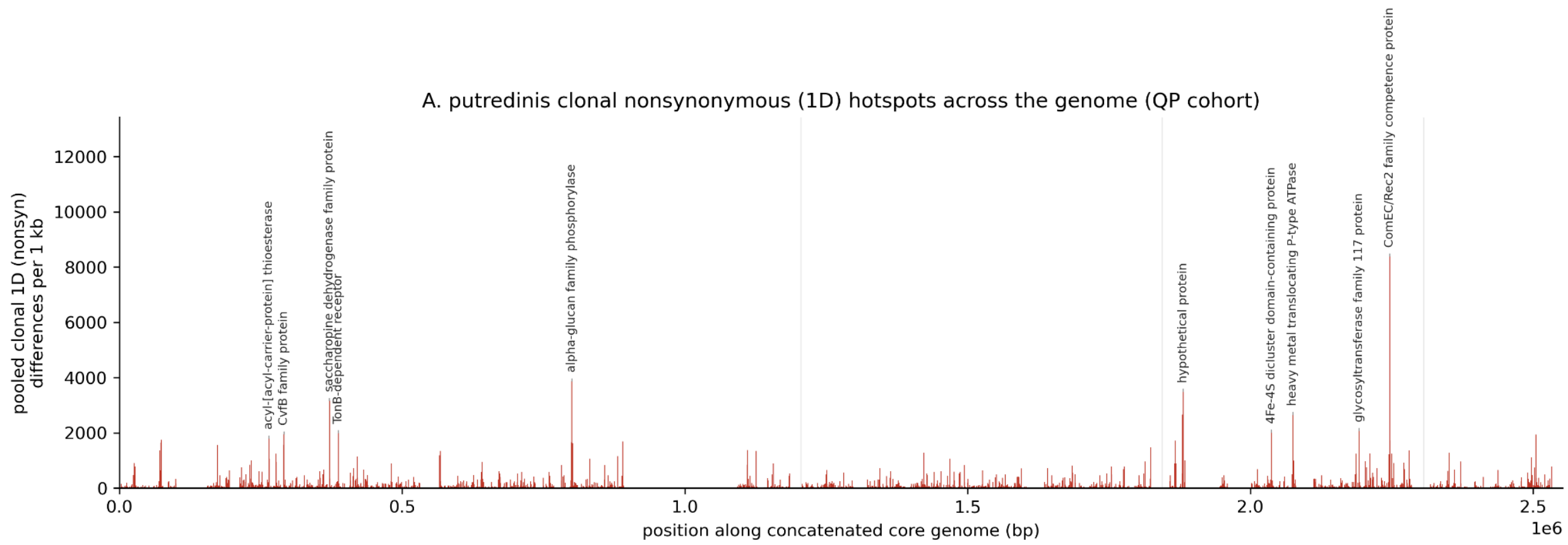

Figure R3.6. Pooled clonal nonsynonymous (1D) differences per 1-kb window along the A. putredinis core genome, summed over all QP close pairs after removing CP-HMM-detected recombination (without boundary extension), with the top hotspots labeled by gene product. Clonal nonsynonymous differences are concentrated at a few loci rather than spread uniformly, and the single tallest hotspot coincides with a ComEC/Rec2-family natural-competence protein.

Phrasing, typos:

(10) It might be worth re-writing (S5) to make it clear that it's a Hill function. I also was at first confused why Fig S1 says k=10, when the log-log slope looks like 4. It was only when I looked at the code that I realized that (S5) was mixing e with log base 10. I'd stick with one base, which would make k more interpretable.

Thanks for pointing this out. As described in our response to comments #2 and #8 above, we have replaced the entire Hill function approach with a nonparametric fit, which should be easier to understand.

(11) 90-91: I'd separate out this inequality into two pieces. $R T_\mathrm{MRCA} \gg 1$ is the condition for recombination to inflate the number of SNVs, while $R T \ll 1$ is the condition for the clonal ancestry to still be significant. The current phrasing makes it sound like both inequalities are relevant for whether recombination can substantially inflate the number of SNPs. Although I guess the whole thing is kind of confusing given that the authors aren't just using a single rate of recombination R.

Thanks for this suggestion – we have revised the text along the lines suggested. (We have also edited the text of this paragraph to emphasize that R may itself depend on T.)

3. 248: "intruiging"
4. 265: cut "comprised of"

Thanks for catching these – we’ve fixed them in the revised manuscript.

Sincerely,
Daniel Weissman
