# Supplementary material for: Recombination shapes the dynamics of selective constraint in global populations of human gut bacteria
Source: PLoS Biol. 2026 Oct 12;24(10):e3004052. doi: 10.1371/journal.pbio.3004052 (PMC13690274; doi:10.1371/journal.pbio.3004052)
Supplement: Supplementary file 3 [file pbio.3004052.s005.pdf]

RESPONSE TO REVIEWERS

I. EDITORIAL REQUESTS:

a) We need your Title to contain an active verb and to encapsultate the findings more explicitly and accessibly (a quick survey of my colleagues suggests that "dN/dS" is not widely understood); please change it accordingly. Looking at your Abstract, I was thinking something like "Recombination in bacterial populations shapes genome-wide patterns of selective constraint"

Thanks for this suggestion. We have modified it slightly: “Recombination shapes the dynamics of selective constraint in global populations of human gut bacteria”.

b) Please address the remaining requests from reviewer #3.

We have addressed these remaining requests – see our point-by-point response below.

c) Please address my Data Policy requests below; specifically, we need you to supply the numerical values underlying Figs 2BCD, 3, 4AB, 5AB, ABCDEFGHIJKLMNOPQR, either as a supplementary data file or as a permanent DOI’d deposition. I note that you already have an associated GitHub deposition (https://github.com/zhiru-liu/dNdS_dynamics), which looks quite thorough. Please could you confirm whether the data and code in this deposition are sufficient to recreate the Figures?

Yes, we have added these numerical values to the associated repositories and have provided a link in the figure legend (item f below).

d) Please re-name your supplementary Figures from A-R to S1-S18.

Following the additional correspondence in our follow-up email, we’ve left these in alphabetical form for now (awaiting further guidance from the copy-edit team).

e) Because Github depositions can be readily changed or deleted, please make a permanent DOI’d copy (e.g. in Zenodo) and provide this URL (see below).

We have added these to the revised manuscript.

f) Please cite the location of the data clearly in all relevant main and supplementary Figure legends, e.g. “The data and code needed to generate this Figure can be found in https://zenodo.org/records/XXXXXXXX”; also update your Data Availability Statement accordingly.

We have added these links to the figure legends.

g) Please include the URLs of your funders in the Financial Disclosure statement.

We have added these to the Financial Disclosure statement.

II. REVIEWERS' COMMENTS:

Reviewer #3:

The authors have fully addressed the points from my previous review. The authors have more than fully addressed the major question in my previous review (whether there was a self-consistent dynamical theory), creating two new simulation models and analyzing them.

I just had one question about these additional simulation results. Is SI Figure R showing the results of both simulation models? Are they indistinguishable from each other? I was expecting to see two sets of points for every value of R / L mu.

Thanks for bringing this up. We set up the figure such that with the appropriate parameter re-scalings, both models can be depicted with a common set of simulations (just with different interpretations of key parameters like R, N, and Tmrca). We discuss the rationale behind this briefly in the corresponding Methods section, but we can see how this wouldn't be obvious from the figure, so we have added some additional text to the caption of SI Figure R to more explicitly point this out:

> **Figure R: Genome-wide simulations of the purifying selection null model generate strong selective interference.** Whole-genome, individual-based simulations of the purifying selection model in Fig. 3 were performed in two simple metapopulation models, corresponding to the limits of pure intra-host or inter-host competition (Methods Section 5). Using standard diffusion scalings, both models can be depicted with the same set of simulation points, but with different interpretations of the underlying parameters. (a) Realized values of T_{mrca} (estimated from the pairwise diversity at linked neutral sites) as a function of the effective population size N. In the pure intra-host competition model, T_{mrca} refers to the local coalescence time within a single person's microbiome, and N is the corresponding intra-host population size. In the pure inter-host competition model, T_{mrca} refers to the global coalescence timescale ($T_{\mathrm{mrca}} \equiv \overline{d_S}/2\mu$), and N corresponds to the global effective population size (see Methods Section 5). Points show simulation results for four different recombination rates R; in the pure intra-host competition model, we only simulated intra-host transfers ($R \approx R_{\mathrm{int}}$), while the inter-host model only considers transfers between strains in different hosts ($R \approx R_{\mathrm{ext}}$). Dashed lines show three different timescales for comparison: the observed

As an extremely minor point, the discussion of the pure intra-host model refers a lot to dN(c) / dS(c) curves, so the authors _might_ want to consider a panel showing them in Figure R. But they have already gone above and beyond with these revisions, so they may not want to slow down publication any more.

Unfortunately, for performance reasons, our simulations were not set up to measure explicit

dN/dS curves in their current form, so we think this would be better left for future work. However, this comment made us realize that the link between our simulated outputs and the clonal dN/dS curves is not as clear as we could be. So we have expanded the text in the Methods section to show how the dNc/dSc curve is connected from our existing measurements of Npfix(-s1), using the model in Section 6:

invisible to natural selection after they fix, and can only be repaired by subsequent reversions. The dN/dS curves in this scenario can therefore be described by the reversion model in Eq. 28 below, which reduces to

$$\frac{d_N}{d_S} \approx \alpha_0 + (1-\alpha_0)\cdot \frac{2Np_{\rm fix}(-s_1)}{1+\frac{p_{\rm fix}(-s_1)}{p_{\rm fix}(+s_1)}} \cdot \frac{1-e^{-\left(1+\frac{p_{\rm fix}(-s_1)}{p_{\rm fix}(+s_1)}\right)\cdot Np_{\rm fix}(+s_1)d_S}}{\left(1+\frac{p_{\rm fix}(-s_1)}{p_{\rm fix}(+s_1)}\right)\cdot Np_{\rm fix}(+s_1)d_S}\,, \qquad (22)$$

in the special case where $\epsilon = 1$, $r_{\rm on} = N\mu p_{\rm fix}(-s_1)$ and $r_{\rm off} = N\mu p_{\rm fix}(+s_1)$. This implies that when $Np_{\rm fix}(-s_1) \gtrsim 0.1$, dN/dS curve will start at an $\mathcal{O}(1)$ value, and then decay on a divergence scale
