# Supplementary material for: Recombination shapes the dynamics of selective constraint in global populations of human gut bacteria
Source: PLoS Biol. 2026 Oct 12;24(10):e3004052. doi: 10.1371/journal.pbio.3004052 (PMC13690274; doi:10.1371/journal.pbio.3004052)
Supplement: Peer review history

# Editor report: Decision Letter 0

**DOI:** 10.1371/journal.pbio.3004052.r001

**Authors:** Roland Roberts

2 Dec 2025

Dear Dr Good,

Thank you for submitting your manuscript entitled "Dynamics of dN/dS within recombining bacterial populations" for consideration as a Research Article by PLOS Biology.

Your manuscript has now been evaluated by the PLOS Biology editorial staff, as well as by an academic editor with relevant expertise, and I'm writing to let you know that we would like to send your submission out for external peer review.

However, before we can send your manuscript to reviewers, we need you to complete your submission by providing the metadata that is required for full assessment. To this end, please login to Editorial Manager where you will find the paper in the 'Submissions Needing Revisions' folder on your homepage. Please click 'Revise Submission' from the Action Links and complete all additional questions in the submission questionnaire.

Once your full submission is complete, your paper will undergo a series of checks in preparation for peer review. After your manuscript has passed the checks it will be sent out for review. To provide the metadata for your submission, please Login to Editorial Manager (https://www.editorialmanager.com/pbiology) within two working days, i.e. by Dec 04 2025 11:59PM.

If your manuscript has been previously peer-reviewed at another journal, PLOS Biology is willing to work with those reviews in order to avoid re-starting the process. Submission of the previous reviews is entirely optional and our ability to use them effectively will depend on the willingness of the previous journal to confirm the content of the reports and share the reviewer identities. Please note that we reserve the right to invite additional reviewers if we consider that additional/independent reviewers are needed, although we aim to avoid this as far as possible. In our experience, working with previous reviews does save time.

If you would like us to consider previous reviewer reports, please edit your cover letter to let us know and include the name of the journal where the work was previously considered and the manuscript ID it was given. In addition, please upload a response to the reviews as a 'Prior Peer Review' file type, which should include the reports in full and a point-by-point reply detailing how you have or plan to address the reviewers' concerns.

During the process of completing your manuscript submission, you will be invited to opt-in to posting your pre-review manuscript as a bioRxiv preprint. Visit http://journals.plos.org/plosbiology/s/preprints for full details. If you consent to posting your current manuscript as a preprint, please upload a single Preprint PDF.

Feel free to email us at plosbiology@plos.org if you have any queries relating to your submission.

Kind regards,

Roli Roberts

Roland Roberts, PhD

Senior Editor

PLOS Biology

rroberts@plos.org

# Peer review: Decision Letter 1

**DOI:** 10.1371/journal.pbio.3004052.r002

**Authors:** Roland Roberts

26 Jan 2026

Dear Dr Good,

Thank you for your patience while your manuscript "Dynamics of dN/dS within recombining bacterial populations" was peer-reviewed at PLOS Biology. It has now been evaluated by the PLOS Biology editors, an Academic Editor with relevant expertise, and by three independent reviewers.

Reviewer #1 calls this “a welcome rigorous approach,” but has a set of concerns regarding the methodological robustness of the bioinformatics which will need to be addressed, plus some statistical questions and presentational suggestions. Reviewer #2 says that they are enthusiastic about the study, but have a few requests for clarification. Reviewer #3 is also positive but has a number of clarifications and suggestions.

In light of the reviews, which you will find at the end of this email, we would like to invite you to revise the work to thoroughly address the reviewers' reports.

Given the extent of revision needed, we cannot make a decision about publication until we have seen the revised manuscript and your response to the reviewers' comments. Your revised manuscript is likely to be sent for further evaluation by all or a subset of the reviewers.

In addition to these revisions, you will need to complete some formatting changes, which you will receive in a follow up email. A member of our team will be in touch with a set of requests shortly.

We expect to receive your revised manuscript within 3 months. Please email us (plosbiology@plos.org) if you have any questions or concerns, or would like to request an extension.

At this stage, your manuscript remains formally under active consideration at our journal; please notify us by email if you do not intend to submit a revision so that we may withdraw it.

**IMPORTANT - SUBMITTING YOUR REVISION**

Your revisions should address the specific points made by each reviewer. Please submit the following files along with your revised manuscript:

1. A 'Response to Reviewers' file - this should detail your responses to the editorial requests, present a point-by-point response to all of the reviewers' comments, and indicate the changes made to the manuscript.

*NOTE: In your point-by-point response to the reviewers, please provide the full context of each review. Do not selectively quote paragraphs or sentences to reply to. The entire set of reviewer comments should be present in full and each specific point should be responded to individually, point by point.

You should also cite any additional relevant literature that has been published since the original submission and mention any additional citations in your response.

2. In addition to a clean copy of the manuscript, please also upload a 'track-changes' version of your manuscript that specifies the edits made. This should be uploaded as a "Revised Article with Changes Highlighted" file type.

*Re-submission Checklist*

When you are ready to resubmit your revised manuscript, please refer to this re-submission checklist: https://plos.io/Biology_Checklist

To submit a revised version of your manuscript, please go to https://www.editorialmanager.com/pbiology/ and log in as an Author. Click the link labelled 'Submissions Needing Revision' where you will find your submission record.

Please make sure to read the following important policies and guidelines while preparing your revision:

*Published Peer Review*

Please note while forming your response, if your article is accepted, you may have the opportunity to make the peer review history publicly available. The record will include editor decision letters (with reviews) and your responses to reviewer comments. If eligible, we will contact you to opt in or out. Please see here for more details:

https://blogs.plos.org/plos/2019/05/plos-journals-now-open-for-published-peer-review/

*PLOS Data Policy*

Please note that as a condition of publication PLOS' data policy (http://journals.plos.org/plosbiology/s/data-availability) requires that you make available all data used to draw the conclusions arrived at in your manuscript. If you have not already done so, you must include any data used in your manuscript either in appropriate repositories, within the body of the manuscript, or as supporting information (N.B. this includes any numerical values that were used to generate graphs, histograms etc.). For an example see here: http://www.plosbiology.org/article/info%3Adoi%2F10.1371%2Fjournal.pbio.1001908#s5

*Blot and Gel Data Policy*

We require the original, uncropped and minimally adjusted images supporting all blot and gel results reported in an article's figures or Supporting Information files. We will require these files before a manuscript can be accepted so please prepare them now, if you have not already uploaded them. Please carefully read our guidelines for how to prepare and upload this data: https://journals.plos.org/plosbiology/s/figures#loc-blot-and-gel-reporting-requirements

*Protocols deposition*

To enhance the reproducibility of your results, we recommend that if applicable you deposit your laboratory protocols in protocols.io, where a protocol can be assigned its own identifier (DOI) such that it can be cited independently in the future. Additionally, PLOS ONE offers an option for publishing peer-reviewed Lab Protocol articles, which describe protocols hosted on protocols.io. Read more information on sharing protocols at https://plos.org/protocols?utm_medium=editorial-email&utm_source=authorletters&utm_campaign=protocols

Thank you again for your submission to our journal. We hope that our editorial process has been constructive thus far, and we welcome your feedback at any time. Please don't hesitate to contact us if you have any questions or comments.

Sincerely,

Roli Roberts

Roland Roberts, PhD

Senior Editor

PLOS Biology

rroberts@plos.org

------------------------------------

REVIEWERS' COMMENTS:

Reviewer #1:

This interesting manuscripts deals with a long-term controversy in molecular evolution/population genetics: the causes of the variation in relative substitution rates of non-synonymous (dN) and synonymous (dS) substitutions. This ratio (dN/dS) is taken as a proxy for the selection regime, dN/dS being in general less than 1, meaning dominance of purifying selection. About two decades ago, it was shown that dN/dS varied with time since the most recent common ancestor (tMRCA), which was suggested to be caused by purifying selection on weakly deleterious mutations. For recent mutations dN/dS is close to 1 (the difference being caused for example by lethal mutations), whereas for distant bacteria dN/dS tends to low values. A couple of years later it was shown that recombination tracts have lower dN/dS which was interpreted as a consequence of higher local tMRCA on the same effect. Meanwhile other hypothesis were published, including the idea that reversion of non-synonymous mutations could explain these trends. This paper presents a welcome rigorous approach to integrate all this in the analysis of bacteria from the gut microbiota. The key results are that recombination vastly accelerates the decrease of dN/dS with time and the mathematical approach provides estimates of key parameters of the population genetics models that underlie the two hypotheses listed above (and this is an important contribution on itself). The paper is thus timely and very relevant. The text is well written and the figures are clear. I have one major technical issue to raise and some minor comments.

- My main concerns are about the bioinformatics analysis. The authors map reads on genomes and use these mappings to identify recombination tracts and compute dN and dS (and the ratio). That poses two types of problems:

- Mapping. Mapping of metagenomics reads is intrinsically error prone, when compared to using assemblies of complete genomes. This is because the latter are usually from pure cultures, are sequenced at more uniform depths, and at high coverage. Metagenomic mapping often leads to inaccuracies inflating the number of changes, which poses a more important problem for comparisons of very closely related genomes (than for the distantly related ones). As a result, this may spuriously inflate the effect observed in this work (low contribution of clonal dN/dS because when polymorphism is rare a lot of it is actually sequencing error)

- Alignment. Usually dN/dS is computed on protein-guided DNA alignments. Not sure that was the case here. That would be best, because from my experience standard DNA alignments, especially in the presence of sequencing errors including frameshifts, inflates dN.

While I don't think the following issues change qualitatively the conclusions, they may change quantitatively the results. And the quantitative aspect is an important part (and contribution) of this work. For example, the strange result for one species if dN/dS =2 does suggest problems in the way reads are assigned to genomes. I suggest the authors take one species from the gut that has enough publicly available complete genomes and compare the two approaches (using metagenomes and genome assemblies). The analysis with Staphylococcus is not satisfactory in this respect because recombination was not assessed as in the other analysis, it's a low recombination bacteria, and is not from the gut (so one cannot compare the genome and metagenomic-based approaches). Using complete genomes would also allow to cover more homogeneously the dS range in the figures (which are very low in the dataset).

-The authors work suggest that dN/dS decrease would be strongly dependent on species recombination rates. This is surely outside of the scope of this paper, but it would be relevant in the future to test this, i.e. pick bacterial species with increasing recombination rates and assess the shapes of decrease in dN/dS.

-There are some statistical biases in the analysis for which I have no great solution, but should be mentioned/discussed. 1) Strictly speaking dS and dN/dS are not statistically independent. This may pose a problem in that random fluctuations in dS in the clonal descent lead to extreme positive values resulting in lower dN/dS. 2) The identification of recombination tracts requires high density of polymorphism. Hence, highly divergent tracts are more likely to be identified than exchanges between very similar sequences. This means two things: the cumulative effect of recombination may be slightly underestimated (because the missing events carry little signa), and the individual effect of recombination events is over-estimated (because the events that are accounted for are the ones with highest levels of polymorphism).

- I suggest to add the methods in the main text, instead of leaving them as supplementary material. That's the (good) standard of PLoS Biology.

- Very minor. In figure 1A I would suggest to have as short term only the adaptive and put a middle-term for neutral. Neutral would be the reference.

- I suggest adding explicitly also the git repertoire for the program to identify recombination using an HMM. It would be useful to the community.

Reviewer #2:

[identify themselves as Nandita Garud and Peter Laurin]

Summary

This paper makes an important contribution to the study of bacterial evolution by showing that the decay of dN / dS can be highly confounded in recombinant bacterial populations. Thus, the time in which we can observe evolution directly acting between clonal descendants may be very short, and the range of the curve informative to evolutionary models may be quite small. Nonetheless, the authors show convincingly that a purifying selection model may be a decisively better fit to the dN / dS curve of species in the human gut microbiome than a recently-proposed model of adaptive reversion. Overall, their findings have broad implications to the interpretation of dN / dS in bacteria and limitations thereof, and provide further insight into the evolution of gut commensals.

We overall are very enthusiastic about this work, and have minor comments/questions for the authors.

1. Can you speculate why the "effective" fitness costs estimated by the decay in dN/dS might differ from those estimated in other methods (e.g. LD, SFS)

2. Given there is such a small amount of curve to fit selection coefficients, it seems that the equilibrium value of dN / dS (i.e. in well-recombined strains) may have a large effect on the estimation. How sensitive is the fit of s/mu to the equilibrium value?

3. Could the authors make clearer what TMRCA is in this paper? Is it the coalescence time of the entire species (e.g. Fig 1A) or the average coalescence time between two genomes? Are they meant to be the same given extensive recombination across strains of the same species? Could the authors provide a little more explanation? Maybe this is well defined in Liu and Good 2024 PloS Biology and requires a small citation here.

4. Line 161: "Fig. 2D shows that the dN/dS levels on the clonal backbone remain close to one even as the nominal dS values span more than three orders of magnitude (10^-5 < dS < 10^-2; Fig. 2D). However, because these genome-wide dS levels are inflated by recombination, they overestimate the true divergence time between closely related strains."

This is a very important point, and critical to understanding Fig 2. The initial lack of decay when plotted by dS confused us on first reading, as well. We recommend adding this context earlier to the Fig 2 section: Quantifying the impact of homologous recombination on dN/dS dynamics because otherwise the intuition that there should be a decay with dS doesn't line up with the data.

5. How were the parameters chosen for the "recombination theory" line in Fig 2B? Is it by visual inspection/fitting from fig S1? If so, then in what sense are there no free parameters with the phenomenological model? But the point is well taken that the dynamics of dN/dS can be described well with the characteristics of recombined fragments alone.

Signed

Nandita Garud and Peter Laurin

Reviewer #3:

[Identifies himself as Daniel Weissman]

In this manuscript, Liu and Good use data from many gut microbiome species and Staphylococcus aureus to show that the observed decay of pairwise dN/dS with genetic distance within species is primarily driven by recombination. I think this is a fascinating result that suggests a way forward for a controversial question. Fig 2 is a striking demonstration of the importance of recombination, and Fig 3 clearly illustrates that the true, slower selective dynamics in non-recombined regions of the genome. I learned a lot from the manuscript.

Major question:

The one big thing that I think could be clarified is the extent to which the authors have a found a full, self-consistent model for the data. A key motivation for this work is Torrillo and Lieberman 2024, which pointed out that the pervasive modest purifying selection inferred from previous dN/dS analyses could lead to Muller's ratchet. In other words, the simple model that described a single locus might break down when applied simultaneously throughout the genome. With the authors' inferred selective coefficients, how frequent would selection have to be and how big would the population need to be to avoid Muller's ratchet? Or am I just totally missing something? 321-331 in the Discussion kind of gets at this, but it's more focused on how to derive the effective model from micro dynamics, whereas I'm curious about the consistency of the effective model itself.

Minor questions:

1. Related to the major comment above, I would add just a sentence or two more emphasizing that the recombination-accumulation function (S5) is just a fit and doesn't come out of any dynamical model, and that it requires _super-linear_ accumulation at short time scales. I guess one may get something like that naturally in the model because selection is promoting recombination events that fix accumulated deleterious mutations, but this isn't something that anybody has worked out, is it? Do the authors think this is an interesting area for future work?

2. Is the spread in dN/dS values around the mean way larger than what would be predicted by naively applying the simple purifying selection model? What about within species?

3. It looks like there's a downward trend in the mean dN/dS values across species, with more diverse species having lower mean dN/dS. Am I fooling myself there? Could that be a trace of truly weakly selected mutations?

4. Would it be easy to add supplemental plots showing the bounds on the cumulative DFE from (S13-14)?

5. When talking about the bounds on s, it's a bit tricky to decide when to leave things in terms of other parameters ("s << 10^5 mu") and when to plug in typical values of those parameters to allow writing absolute numbers ("s << 10^-4"). In the first paragraph of the Discussion, I think it might be nice to add a line turning the lower bound s \gtrsim 1/TMRCA into absolute numbers to complement the s << 10^-4 upper bound.

6. I found 341-343 hard to understand. I think 954-967 in the supplement explains what the authors are saying there. Perhaps a chunk of that paragraph could be moved up to the Discussion?

7. It would be nice to have a curve showing some kind of simple binned or smoothed average of the data in Fig S1.

8. I'd invite the authors to feel free to throw in a line of wild speculation about Alistipes putredinis if they want.

Phrasing, typos:

1. It might be worth re-writing (S5) to make it clear that it's a Hill function. I also was at first confused why Fig S1 says k=10, when the log-log slope looks like 4. It was only when I looked at the code that I realized that (S5) was mixing e with log base 10. I'd stick with one base, which would make k more interpretable.

2. 90-91: I'd separate out this inequality into two pieces. $R T_\mathrm{MRCA} \gg 1$ is the condition for recombination to inflate the number of SNVs, while $R T \ll 1$ is the condition for the clonal ancestry to still be significant. The current phrasing makes it sound like both inequalities are relevant for whether recombination can substantially inflate the number of SNPs. Although I guess the whole thing is kind of confusing given that the authors aren't just using a single rate of recombination R.

3. 248: "intruiging"

4. 265: cut "comprised of"

Sincerely,

Daniel Weissman

# Author comment: Author response to Decision Letter 2

**DOI:** 10.1371/journal.pbio.3004052.r003

30 Jul 2026

Attachment Submitted filename: response_to_reviewers.pdf

# Peer review: Decision Letter 2

**DOI:** 10.1371/journal.pbio.3004052.r004

**Authors:** Roland Roberts

8 Sep 2026

Dear Ben,

Thank you for your patience while we considered your revised manuscript "Dynamics of dN/dS within recombining bacterial populations" for publication as a Research Article at PLOS Biology. This revised version of your manuscript has been evaluated by the PLOS Biology editors, the Academic Editor and the original reviewers.

Based on the reviews, we are likely to accept this manuscript for publication, provided you satisfactorily address the remaining points raised by the reviewers and the following data and other policy-related requests.

IMPORTANT - please attend to the following:

a) We need your Title to contain an active verb and to encapsultate the findings more explicitly and accessibly (a quick survey of my colleagues suggests that "dN/dS" is not widely understood); please change it accordingly. Looking at your Abstract, I was thinking something like "Recombination in bacterial populations shapes genome-wide patterns of selective constraint"

b) Please address the remaining requests from reviewer #3.

c) Please address my Data Policy requests below; specifically, we need you to supply the numerical values underlying Figs 2BCD, 3, 4AB, 5AB, ABCDEFGHIJKLMNOPQR, either as a supplementary data file or as a permanent DOI’d deposition. I note that you already have an associated GitHub deposition (https://github.com/zhiru-liu/dNdS_dynamics), which looks quite thorough. Please could you confirm whether the data and code in this deposition are sufficient to recreate the Figures?

d) Please re-name your supplementary Figures from A-R to S1-S18.

e) Because Github depositions can be readily changed or deleted, please make a permanent DOI’d copy (e.g. in Zenodo) and provide this URL (see below).

f) Please cite the location of the data clearly in all relevant main and supplementary Figure legends, e.g. “The data and code needed to generate this Figure can be found in https://zenodo.org/records/XXXXXXXX”; also update your Data Availability Statement accordingly.

g) Please include the URLs of your funders in the Financial Disclosure statement.

As you address these items, please take this last chance to review your reference list to ensure that it is complete and correct. If you have cited papers that have been retracted, please include the rationale for doing so in the manuscript text, or remove these references and replace them with relevant current references. Any changes to the reference list should be mentioned in the cover letter that accompanies your revised manuscript.

In addition to these revisions, you may need to complete some formatting changes, which you will receive in a follow up email. A member of our team will be in touch with a set of requests shortly. If you do not receive a separate email within a few days, please assume that checks have been completed, and no additional changes are required.

We expect to receive your revised manuscript within two weeks.

To submit your revision, please go to https://www.editorialmanager.com/pbiology/ and log in as an Author. Click the link labelled 'Submissions Needing Revision' to find your submission record. Your revised submission must include the following:

- a cover letter that should detail your responses to any editorial requests, if applicable, and whether changes have been made to the reference list

- a Response to Reviewers file that provides a detailed response to the reviewers' comments (if applicable, if not applicable please do not delete your existing 'Response to Reviewers' file.)

- a track-changes file indicating any changes that you have made to the manuscript.

NOTE: If Supporting Information files are included with your article, note that these are not copyedited and will be published as they are submitted. Please ensure that these files are legible and of high quality (at least 300 dpi) in an easily accessible file format. For this reason, please be aware that any references listed in an SI file will not be indexed. For more information, see our Supporting Information guidelines:

https://journals.plos.org/plosbiology/s/supporting-information

*Published Peer Review History*

Please note that you may have the opportunity to make the peer review history publicly available. The record will include editor decision letters (with reviews) and your responses to reviewer comments. If eligible, we will contact you to opt in or out. Please see here for more details:

https://plos.org/published-peer-review-history/

*Press*

Should you, your institution's press office or the journal office choose to press release your paper, please ensure you have opted out of Early Article Posting on the submission form. We ask that you notify us as soon as possible if you or your institution is planning to press release the article.

*Protocols deposition*

To enhance the reproducibility of your results, we recommend that if applicable you deposit your laboratory protocols in protocols.io, where a protocol can be assigned its own identifier (DOI) such that it can be cited independently in the future. Additionally, PLOS ONE offers an option for publishing peer-reviewed Lab Protocol articles, which describe protocols hosted on protocols.io. Read more information on sharing protocols at https://plos.org/protocols?utm_medium=editorial-email&utm_source=authorletters&utm_campaign=protocols

Please do not hesitate to contact me should you have any questions.

Sincerely,

Roli

Roland Roberts, PhD

Senior Editor

rroberts@plos.org

PLOS Biology

------------------------------------------------------------------------

DATA POLICY:

You may be aware of the PLOS Data Policy, which requires that all data be made available without restriction: http://journals.plos.org/plosbiology/s/data-availability. For more information, please also see this editorial: http://dx.doi.org/10.1371/journal.pbio.1001797

Note that we do not require all raw data. Rather, we ask that all individual quantitative observations that underlie the data summarized in the figures and results of your paper be made available in one of the following forms:

1) Supplementary files (e.g., excel). Please ensure that all data files are uploaded as 'Supporting Information' and are invariably referred to (in the manuscript, figure legends, and the Description field when uploading your files) using the following format verbatim: S1 Data, S2 Data, etc. Multiple panels of a single or even several figures can be included as multiple sheets in one excel file that is saved using exactly the following convention: S1_Data.xlsx (using an underscore).

2) Deposition in a publicly available repository. Please also provide the accession code or a reviewer link so that we may view your data before publication.

Regardless of the method selected, please ensure that you provide the individual numerical values that underlie the summary data displayed in the following figure panels as they are essential for readers to assess your analysis and to reproduce it: Figs 2BCD, 3, 4AB, 5AB, ABCDEFGHIJKLMNOPQR. NOTE: the numerical data provided should include all replicates AND the way in which the plotted mean and errors were derived (it should not present only the mean/average values).

IMPORTANT: Please also ensure that figure legends in your manuscript include information on where the underlying data can be found, and ensure your supplemental data file/s has a legend.

Please ensure that your Data Statement in the submission system accurately describes where your data can be found.

------------------------------------------------------------------------

CODE POLICY

Per journal policy, if you have generated any custom code during the course of this investigation, please make it available without restrictions. Please ensure that the code is sufficiently well documented and reusable, and that your Data Statement in the Editorial Manager submission system accurately describes where your code can be found. More information on our Code Policy, what and how to share can be found here: https://journals.plos.org/plosbiology/s/code-availability

Please note that we cannot accept sole deposition of code in GitHub, as this could be changed after publication. However, you can archive this version of your publicly available GitHub code to Zenodo. Once you do this, it will generate a DOI number, which you will need to provide in the Data Accessibility Statement (you are welcome to also provide the GitHub access information). See the process for doing this here: https://docs.github.com/en/repositories/archiving-a-github-repository/referencing-and-citing-content

------------------------------------------------------------------------

REVIEWERS' COMMENTS:

Reviewer #1:

I thank the authors for the very detailed answers to my queries and suggestions. Overall, I think they made a great job and with the new changes I'm now fully convinced. I think this is a very interesting contribution that will spur further works bringing new light on how recombination affects natural selection in bacteria. Congratulations. I have no other comments.

Reviewer #2:

The authors have thoroughly addressed all review comments.

Reviewer #3:

The authors have fully addressed the points from my previous review. The authors have more than fully addressed the major question in my previous review (whether there was a self-consistent dynamical theory), creating two new simulation models and analyzing them.

I just had one question about these additional simulation results. Is SI Figure R showing the results of both simulation models? Are they indistinguishable from each other? I was expecting to see two sets of points for every value of R / L mu.

As an extremely minor point, the discussion of the pure intra-host model refers a lot to dN(c) / dS(c) curves, so the authors _might_ want to consider a panel showing them in Figure R. But they have already gone above and beyond with these revisions, so they may not want to slow down publication any more.

# Author comment: Author response to Decision Letter 3

**DOI:** 10.1371/journal.pbio.3004052.r005

21 Sep 2026

Attachment Submitted filename: response_to_reviewers_auresp_3.pdf

# Editor report: Decision Letter 3

**DOI:** 10.1371/journal.pbio.3004052.r006

**Authors:** Roland Roberts

28 Sep 2026

Dear Dr Good,

I hope this e-mail finds you well. I am Melissa Vázquez Hernández and I am writing on behalf of my colleague Dr. Roli Roberts.

Thank you for the submission of your revised Research Article "Recombination shapes the dynamics of selective constraint in global populations of human gut bacteria" for publication in PLOS Biology. On behalf of my colleagues and the Academic Editor, Deepa Agashe, I am pleased to say that we can in principle accept your manuscript for publication, provided you address any remaining formatting and reporting issues. These will be detailed in an email you should receive within 2-3 business days from our colleagues in the journal operations team; no action is required from you until then. Please note that we will not be able to formally accept your manuscript and schedule it for publication until you have completed any requested changes.

Please take a minute to log into Editorial Manager at http://www.editorialmanager.com/pbiology/, click the "Update My Information" link at the top of the page, and update your user information to ensure an efficient production process.

PRESS

We frequently collaborate with press offices. If your institution or institutions have a press office, please notify them about your upcoming paper at this point, to enable them to help maximise its impact. If the press office is planning to promote your findings, we would be grateful if they could coordinate with biologypress@plos.org. If you have previously opted in to the early version process, we ask that you notify us immediately of any press plans so that we may opt out on your behalf.

We also ask that you take this opportunity to read our Embargo Policy regarding the discussion, promotion and media coverage of work that is yet to be published by PLOS. As your manuscript is not yet published, it is bound by the conditions of our Embargo Policy. Please be aware that this policy is in place both to ensure that any press coverage of your article is fully substantiated and to provide a direct link between such coverage and the published work. For full details of our Embargo Policy, please visit http://www.plos.org/about/media-inquiries/embargo-policy/.

Thank you again for choosing PLOS Biology for publication and supporting Open Access publishing. We look forward to publishing your study.

Sincerely,

Melissa

Melissa Vázquez Hernández, PhD

Associate Editor

PLOS Biology

mvazquezhernandez@plos.org

on behalf of

Roland G Roberts, PhD, PhD

Senior Editor

PLOS Biology

rroberts@plos.org
